# The Structural Characterization and Immunomodulatory Activity of Polysaccharides from *Pleurotus abieticola* Fruiting Bodies

**DOI:** 10.3390/nu14204410

**Published:** 2022-10-20

**Authors:** Meichen Pan, Fange Kong, Lei Xing, Lan Yao, Yu Li, Yang Liu, Changtian Li, Lanzhou Li

**Affiliations:** Engineering Research Center of Chinese Ministry of Education for Edible and Medicinal Fungi, Jilin Agricultural University, Changchun 130118, China

**Keywords:** *Pleurotus abieticola*, polysaccharide, immunomodulatory effect, structural analysis, intestinal microflora, Nrf2

## Abstract

Polysaccharides obtained from mushrooms have been reported to possess immunomodulatory properties. In this study, a water-soluble polysaccharide was purified from the fruiting bodies of *Pleurotus abieticola*, entitled PAPS1. After its composition and structural analysis, the immunomodulatory activity was investigated in immunosuppressed mice induced by cyclophosphamide (CTX) at a dosage of 70 mg/kg by intraperitoneal injection for 7 days. After 28 days of intragastric administration, PAPS1 alleviated cyclophosphamide (CTX)-induced histopathological damage and increased the expressions of splenic CD4, CD8, CD56 and IgM in the serums of immunosuppressed mice. PAPS1 suppressed the oxidative stress indicated by preventing the increases in ROS and MDA levels. According to the intestinal microflora analysis, PAPS1 regulated 11 bacteria at the gene level, including *Helicobacter* and *Paraprevotella*, which are related to immunity and oxidative capacity. Compared with CTX-treated mice, significant increases in immune-related cytokines, such as interleukin (IL)-2, IL-6 and IL-12 in the serums of mice treated with PAPS1, were observed. Finally, PAPS1 can strongly increase the expression of nuclear factor erythroid 2-related factor 2 (Nrf2) and its downstream proteins. In conclusion, PAPS1-boosted immunity may be related to its suppression on oxidative stress via enhancing the activity of Nrf2 signaling. Thus, PAPS1 can be investigated as a candidate for immunomodulatory therapy.

## 1. Introduction

Mushrooms are not only utilized as food, but they are also used for health purposes. Due to the long history of medicinal use in China, approximately 200 species of mushrooms have been cultivated here, with cultivation being the fifth largest agricultural sector of this country [1]. Mushrooms contain bioactive compounds, such as polysaccharides and proteins, which possess strong immunomodulatory properties [2]. For example, polysaccharides isolated from *Ganoderma sinense* with a molecular weight of 32 kDa and a backbone of (1→4)- and (1→6)-Glc*p* induce B cell proliferation in BALB/c mice, thus enhancing immunity [3]. In contrast, hypoimmunity leads to a significantly increased risk of invasion by pathogens as well as autoimmune diseases. Chemotherapy, an important means for cancer therapy, has immunosuppressive properties, causing bone marrow suppression and gonadal toxicity [4]. Cyclophosphamide (CTX) is one of the most widely used alkylating cytotoxic drugs for the treatment of several cancers. CTX used in high dosages can lead to severe side effects, such as immunosuppression, including a reduction in body weight, macrophage phagocytosis and natural killer (NK) cell activity [5].

Commensal microbiota have a highly co-evolved relationship with the immune system. The lack of gut microbiota leads to significant immune system deficiency [6]. The colonization of gnotobiotic mice with Clostridial strains can enhance the adaptive immune system by anti-inflammatory-induced Tregs or interleukin (IL)-10 expression [7]. *Bacteroides fragilis* also can induce an IL-10 response in intestinal T cells by recognizing polysaccharide-A, thereby preventing the mucosal barrier from damage [8]. An interesting study showed that treatment with prebiotics causes a significant increase in immunoglobulin A (IgA) in feces and a significant reduction in interferon γ, human-granulocyte-macrophage-colony-stimulating factor and IL-1β expressions, indicating the regulation of the immune balance of the gut microbiome [9]. Moreover, intestinal microbiota directly interact with immune cells through antigen presentation to naïve T cells, or they regulate host immunity by secreting metabolites with immunomodulatory properties, such as short-chain fatty acids (SCFA) [10]. According to clinical data, these metabolites determine immune enhancement by counteracting oxidative stress and increasing the expressions of CD4 or CD8 in T cells [11].

Recently, natural polysaccharides with immunomodulatory properties have gained increased attention. Lentinan, with a triple helical structure of β-(1→3)-D-glucan, can bind to dectin-1 mainly through hydrogen bonds and CH-π interactions, leading to strong immune enhancement [12]. Long or short-chain polymers of glucose subunits rich in β-glucans, found in mushrooms, possess immunomodulatory and anti-inflammatory effects [13]. As mentioned above, *Ganoderma leucocontextum* polysaccharides, containing a backbone of →4)-α-D-Glc*p*-(1→4,6)-β-D-Glc*p*-(1→ linked with a β-Glc*p*-(1→ branch, exert immunomodulatory effects by activating NF-κB signaling in RAW 264.7 macrophages [14].

*Pleurotus* spp. are a group of edible ligninolytic mushrooms with medicinal and nutritional properties. *Pleurotus ostreatus* polysaccharides increase the cytotoxicity of NK cells against tumor cells such as Dalton’s lymphoma cells [15]. *Pleurotus eryngii* polysaccharides containing a 3-O methylated mannogalactan reduce the levels of nitric oxide and cytokines in interferon-γ-induced D2SC/1 dendritic cells [16]. However, few studies have focused on *Pleurotus abieticola (P. abieticola)*, originally found in northeastern and northwestern China and in the Russian Far East [17,18]. Currently, *P. abieticola* is successfully cultivated on nematode-infected *Pinus massoniana* chips [19]. The few reports referring to its bioactive components and medicinal properties strongly limit its application both as a food and as a medicine.

Our purpose was to identify the polysaccharides contained in the cultured fruiting bodies of *P. abieticola* and to investigate their potential medicinal value. We identified, isolated and purified water-soluble PA polysaccharides (PAPS1) and characterized their physicochemical properties, including molecular weight, monosaccharide composition, glycosidic linkage and chain conformation. We investigated their immunomodulatory effects, using a CTX-induced immunosuppression model in mice, and the implications of nuclear factor erythroid 2-related factor 2 (Nrf2) signaling in mediating these effects.

## 2. Materials and Methods

### 2.1. Isolation and Purification of Polysaccharides from P. abieticola

*P. abieticola* (PA) fruiting bodies were provided and identified by Prof. Li Yu from the Jilin Agricultural University (Changchun, Jilin Province, China). They were dried at 40–50 °C, pulverized and stored in a dry environment. 

#### 2.1.1. Extraction of Polysaccharides

According to our previous work [20] and extraction condition results, the method for PAPS1 preparation was as follows. The powder obtained from the PA fruiting bodies was extracted twice with double-distilled (D.D.) water at a ratio of 1:30 (*w*/*v*). Each time, the mixture was kept at 60 °C for 2 h. Supernatants were collected and concentrated. An aqueous solution of ethanol (final concentration 80% (*v*/*v*) was added to the supernatant for 24 h. After centrifugation for 10 min, the precipitate was isolated and treated with Savage reagent (4:1 chloroform: n-butanol, *v*/*v*), and the mixture was stirred for 30 min to remove the proteins six times. The deproteinized solution was lyophilized after dialysis against D.D. water at 4 °C for 48 h. The D.D. water was changed every 6 h.

#### 2.1.2. Purification of Polysaccharides

The lyophilizate was dissolved with D.D. water and was applied to a diethylaminoethylcellulose-52 (DEAE-52) anion exchange column (4 × 60 cm; C8930, Beijing Solarbio Science & Technology Co., Ltd., Beijing, China). The column was eluted with distilled water, followed by 0.1, 0.3 and 0.5 M NaCl solutions at 1.0 mL/min to isolate the polysaccharides. HiPrep 26/60 Sephacryl S-400 High Resolution (HR) (2.6 × 60 cm; 28-9356-05, Danaher Corporation, Washington, WA, USA) and HiLoad 16/600 Superdex 200 prep grade column (1.6 × 60 cm; 28-9893-35, GE Healthcare, Uppsala, Sweden) were used for further purification. The concentration of the collected PAPS1 was measured using the phenol-sulfuric acid method [21].

### 2.2. Composition and Structural Analysis of PAPS1

#### 2.2.1. Composition Analysis of PAPS1

Similar to our previous study [20], the total sugar and protein contents were detected by phenol–sulfuric acid colorimetry and a bicinchoninic acid (BCA) assay (23225, Thermo Scientific, Carlsbad, CA, USA), respectively.

#### 2.2.2. Ultraviolet-Visible (UV-Vis) Analysis of PAPS1

The UV-Vis spectrum of PAPS1 was obtained using a multifunctional enzyme labeling instrument (1510-04201, Thermo Fisher Scientific, Waltham, MA, USA) at the wavelength range of 200 to 800 nm. 

#### 2.2.3. Molecular Weight (Mw) and Homogeneity Analysis of PAPS1

The Mw and homogeneity of PAPS1 were detected by light scattering gel permeation chromatography coupled online to a multi-angle laser light scattering (GPC-MALLS) system equipped with the Gel exclusion chromatography column (Ohpak SB-806 HQ, 300 × 8 mm), the differential detector (Optilab T-rEX, Wyatt technology, Santa Clara, CA, USA) and a laser light-scattering detector (DAWN HELEOS II, Wyatt technology) were used to determine the homogeneity and the molecular weight. The detection conditions were as follows: the column oven temperature was 40 °C; the injection volume was 500 μL; the mobile phase was 0.1 M NaNO_3_; the flow rate was 1.0 mL/min; and the isocratic and gradient elution duration was 35 min.

#### 2.2.4. Monosaccharide Composition Analysis of PAPS1

The monosaccharide components of hydrolyzed PAPS1 were detected using a high-performance liquid chromatography (HPLC) system (ICS500^+^, Thermo Fisher Scientific, USA) equipped with a liquid chromatography (LC) column (150 × 3.0 mm, 10 μm, Dionex™ CarboPac™ PA20) and an electrochemical detector. The injection volume was 5.0 μL. The flow rate was set to 0.5 mL/min for mobile phase A (0.1 M NaOH) and B (0.1 M NaOH, 0.2 M NaAc). The column temperature was 30 °C. 

#### 2.2.5. Methylation Analysis of PAPS1

The methylation and acetylation reaction analyses were performed using gas chromatography–mass spectrometry (GC-MS) (Agilent Technologies Inc., Santa Clara, CA, USA). The analytical procedures [20] are detailed in the Supplementary Methods. 

#### 2.2.6. Nuclear Magnetic Resonance (NMR) Analysis of PAPS1

NMR measurements were obtained using a Bruker Avance AV600 NMR spectrometer (Rheinstetten, Germany) at 500 and 126 MHz for ^1^H and ^13^C, respectively. The analytical procedures [22] are detailed in the Supplementary Methods.

### 2.3. Animal Immunosuppression Model and Agent Administration

The animal experimental protocol used in this study was approved by the Institutional Animal Ethics Committee of Jilin Agricultural University (approval No. 2021-06-03-001, approval date 3 June 2021) and was carried out according to the institutional guidelines. A total of 40 male BALB/c mice (6–8 weeks old, 18–22 g, specific pathogen-free grade) were purchased from Beijing Huafukang Biotechnology Co., Ltd. (Beijing, China) (SCXK (JING) -2019-0008). The mice were kept in a controlled environment (temperature, 23 ± 1 °C; humidity, 50 ± 10%) on a 12 h dark/12 h light cycle (8:00 a.m.–8:00 p.m.). Food and water were provided *ad libitum*. 

After 1 week of acclimatization, 30 mice were administered CTX (Sigma-Aldrich, St. Louis, MO, USA) 70 mg/kg by intraperitoneal injection (i.p.) for 7 days. The CTX was dissolved in normal saline (NS). The immunosuppressed mice were divided randomly into three equal groups, which were intragastrically administered with D.D. water (10 mL/kg) (serving as CTX-only treated mice, *n* = 10); 100 mg/kg PAPS1 (dissolved in D.D. water) (*n* = 10) or 6 mg/kg of lentinan (LNT) (Wuhan DIAO Pharmaceutical Co., Ltd., Wuhan, China) (dissolved in D.D. water) (*n* = 10), once per day for 4 weeks. To avoid restoration of immunity, these mice were injected with CTX (70 mg/kg, i.p.) once a week for the entire duration of the experiment. The control group (*n* = 10) received NS instead of CTX, using the same schedule of administration. The body weights of the mice were recorded daily. After the last administration, blood was collected from the caudal vein and stored in a refrigerator at −80 °C. The mice were euthanized with pentobarbital sodium 100 mg/kg, administered by intraperitoneal injection. The spleens, kidneys, thymuses and livers were collected immediately. A total of 10 mice were randomly divided into two parts for biochemical indices (*n* = 6) and histopathological analysis (*n* = 4).

### 2.4. Histopathological Analysis

#### 2.4.1. Hematoxylin and Eosin (H&E) Staining

Four randomly selected spleens, livers, thymuses and kidneys of each group were fixed with 4% fixative solution at 4 °C for 48 h, embedded in paraffin and then cut into 5 µm-thick slices. The slices were stained with H&E as described previously [23]. The sections were observed under a microscope (BX51; Olympus, Tokyo, Japan).

#### 2.4.2. Immunohistochemistry Staining

Performed as described in our previous work [24], the slides of spleen tissue were repaired by incubating with 3% hydrogen peroxide for further immunohistochemistry. Microscopy (BX51; Olympus, Japan) was used to observe and photograph the tissue with immunoperoxidase staining. The information of primary antibodies is shown in Table S1.

### 2.5. Assessment of Biochemical Indices

Six randomly selected spleen samples were collected from the groups, were homogenized in a radio-immunoprecipitation assay buffer supplemented with 1% protease inhibitor cocktail and 2% phenylmethanesulfonyl fluoride (Sigma-Aldrich). The protein concentration was determined using the BCA Protein Assay Kit (23227; Thermo Fisher Scientific). Biochemical indices were determined using an enzyme-linked immunosorbent assay (ELISA) according to the manufacturer’s instructions. Information on the related ELISA kits of the biochemical indices is shown in Table S2.

### 2.6. Intestinal Microflora Analysis

After the last administration, 4 mice were randomly selected from each group. Four samples of cecal contents from each group were collected immediately after sacrifice. The analysis of the intestinal microflora was performed by 16S rRNA sequencing, on the Illumina MiSeq platform using the MiSeq Reagent Kit v3 (Shanghai Personal Biotechnology Co., Ltd., Shanghai, China) as previously published [25]. Alpha diversity was analyzed using the following indices: Chao1, Faith’s phylogenetic diversity, Good’s coverage, Shannon’s index, Simpson’s index, Pielou’s evenness and the observed species index. The differences were analyzed with Quantitative Insights Into Microbial Ecology version 2 (2019.4), employing the Kruskal–Wallis rank-sum test and Dunn’s test as post hoc tests. Beta diversity was assessed by the non-metric multidimensional scaling (NMDS) ordination of Bray–Curtis matrices. 

### 2.7. Statistical Analysis

Data are presented as the mean ± standard deviation (S.D.). Biochemical indices were calculated and compared between different groups using a one-way ANOVA followed by Dunn’s multiple comparisons post hoc test, using GraphPad Prism 9.0.0 (GraphPad Software Inc., San Diego, CA, USA). Statistical significance was set as a *p* value less than 0.05.

## 3. Results

### 3.1. Purification and Composition Analysis of PAPS1

**Figure 1:**
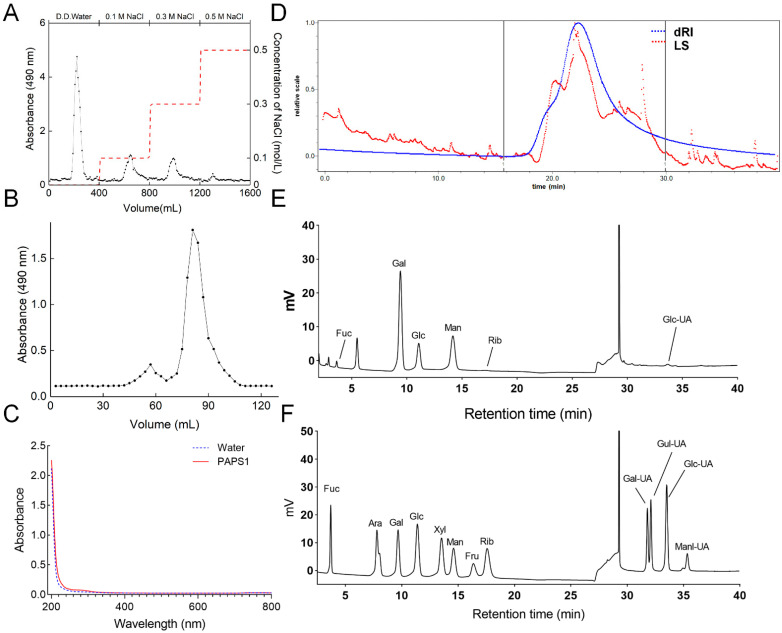
(**A**) Elution profile of the extract of PA fruiting bodies using DEAE-52. Red dotted line represented the 0.1, 0.3 and 0.5 M NaCl solutions, respectively. (**B**) Elution profile of PAPS1 purified by Superdex 200 prep grade column. (**C**) The UV-Vis spectrum of PAPS1 in the wavelength range of 200 to 800 nm. (**D**) Molecular weight analysis of PAPS1. The blue line is the response of the refractive index detector, and the red line is the response of laser light scattering. The calibration curve of pullulan markers is shown in the chromatogram. (**E**) Monosaccharide composition of PAPS1. (**F**) Standard monosaccharide mixture. PA, *Pleurotus abieticola;* PAPS1, water-soluble PA polysaccharides; dRI, differential refractive index; LS, light scattering, Fuc, fucose; Ara, arabinose; Gal, galactose; Glc, glucose; Xyl, xylose; Man, mannose; Fru, fructose; Rib, ribose; Gal-UA, galacturonic acid; Gul-UA, guluronic acid; Glc-UA, glucuronic acid; Manl-UA, Mannuronic acid.

PAPS1 was purified using DEAE Sepharose Fast Flow with D.D. water (Figure 1A), followed by supplementary purification with Sephacryl S-400 HR and Superdex 200 (Figure 1B). The UV-Vis spectrum of PAPS1 showed no UV absorption at 260 nm and 280 nm (Figure 1C), indicating that PAPS1 does not contain proteins or nucleic acids. The carbohydrate content was 0.94 g/g. The radio of Mw/Mn was 1.104, indicating good homogeneity of PAPS1 with an Mw of approximately 17.16 kDa (Figure 1D and Table S3). The main monosaccharides in its composition were fucose (Fuc), galactose (Gal), glucose (Glc) and mannose (Man) with mole percentages of 1.73%, 49.66%, 12.00% and 36.60%, respectively (Figure 1E).

### 3.2. Structural Characterization of PAPS1

The binding ion signals and the types were obtained from the methylation GC–MS chart of PAPS1 (Figure S1) combined with the mass spectrometry database. The attribution results are shown in Table 1. The presence of t-Man(*p*) indicated that PAPS1 has a branched structure.

**Figure 2:**
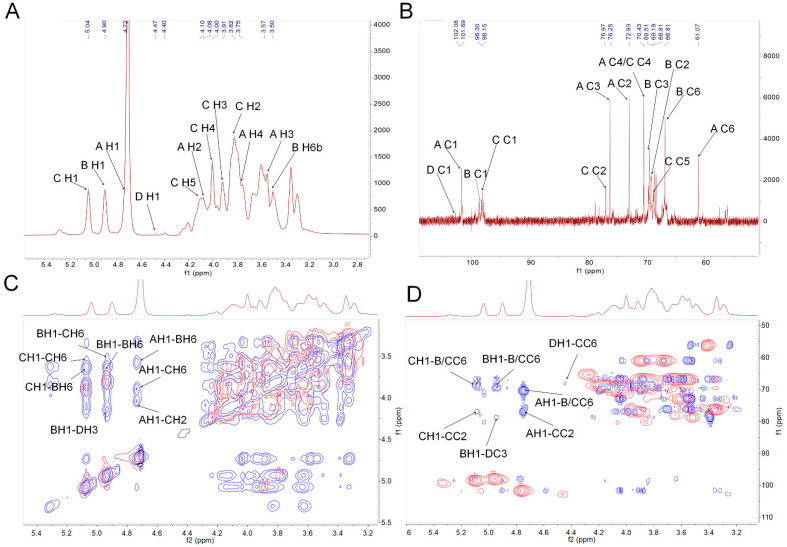
Structural characterization of PAPS1. One-dimensional NMR atlas of (**A**) ^1^H, (**B**) ^13^C NMR, and overlapped two-dimensional NMR atlas of (**C**) ^1^H-^1^H COSY (red) and ^1^H-^1^H NOESY (blue) (COSY-NOESY) and (**D**) HSQC (red) and HMBC (blue) (HSQC-HMBC). PAPS1, water-soluble PA polysaccharides, NMR, Nuclear magnetic resonance; COSY, correlation spectroscopy; NOESY, nuclear overhauser effect spectroscopy; COSY-NOESY, the picture of COSY and NOESY overlapped in the same figure; HSQC, heteronuclear single quantum correlation; HMBC, heteronuclear multiple bond correlation; HSQC-HMBC, the picture of HSQC and HMBC overlapped in the same figure.

In the ^1^H spectrum, the peaks at 4.72, 4.90, 5.04 and 4.47 ppm were assigned to the H-1 of residues A, B, C and D (Figure 2A and Table 2), respectively. Peaks belonging to those protons on sugar rings in a range of 3.0–4.4 ppm were assigned, as shown in Figure S2A and Table 2. In the ^13^C spectrum, anomeric carbon signals at 101.69, 98.30, 98.15 and 102.08 ppm were assigned to the C-1 of residues A, B, C and D (Figure 2B and Table 2), respectively.

According to the literature reports and combined with the results above, the ^1^H and ^13^C chemical shift signals of the residues were assigned, and the results are shown in Table 2. The cross peak at 4.72/101.69 ppm belonged to the H1/C1 of residue A. Combined with ^1^H-^1^H correlation spectroscopy (COSY), the signals at 4.08, 3.57, 3.75, 3.48, 3.85 and 3.66 ppm could be assigned to H2-H6a/b (Figure S2A), respectively. The signals of the C1–C6 of residue A could be confirmed according to the cross peaks in the heteronuclear single quantum correlation (HSQC) spectrum (Figure S2B). The results of other residues similar to residue A were also observed. Consistent with the monosaccharide composition and methylation analysis, PAPS1 contained four major residues: β-D-Man*p*-(1→ (residue A), →6)-α-D-Gal*p*-(1→ (residue B), →2,6)-α-D-Gal*p*-(1→ (residue C) and →3)-β-D-Glc*p*-(1→ (residue D). The proton–proton and proton–carbon single bond correlations were confirmed by the combined results of COSY and nuclear overhauser effect spectroscopy (NOESY) spectrum (COSY-NOESY) (Figure 2C), HSQC and heteronuclear multiple bond correlation (HMBC) (HSQC–HMBC) (Figure 2D), respectively. The anomeric proton H-1 of residue C showed a strong cross signal with its H-6 (5.04/3.85 ppm) (Figure 2C) and its C-6 (5.04/66.78 ppm) (Figure 2D), indicating the presence of 2, 6C1→6, 2C1. In addition, the H-1 of residue C also had strong cross signals with the C-6 (5.04/66.82 ppm) of residue B, which indicated the presences of 2, 6C1→6B1. The H-1 of residue B had a strong cross signal with the H-6 and C-6 of residue C, indicating the existence of 6B1→6, 2C1. The H-1 of residue B had correlated peaks with its H-6 and C-6, indicating the existence of repeating →B→ units. The coupling signals belonged to the H-1 of residue A and the H-2, H-6, C-2 and C-6 of residue C, as shown in HSQC–HMBC (Figure 2D) and COSY–NOESY (Figure 2C), respectively. These results indicated that residue A could be connected to the C-2 of residue C as a branch. Correlated peaks belonged to the H-1 of residue A and the C-6 of residue B, suggesting that residue A not only exists as a branch chain alone, but it also binds to the C-6 of residue B. Furthermore, the H-1 of residue D showed a strong correlated signal with the C-6 of residue C in HSQC–HMBC (Figure 2D).

To summarize, PAPS1 had a main backbone containing → 2,6)-α-D-Gal*p*-(1→, → 6)-α-D-Gal*p*-(1→ and → 3)-β-D-Glc*p*-(1→ residues, and branches mainly with β-D-Man*p*-(1→ and β-D-Man*p*-(1→ 6)-α-D-Gal*p*-(1→ and linkages were attached at the C-2 of the → 2,6)-α-D-Gal*p*-(1→ residue.

### 3.3. Immunoregulatory Effects of PAPS1 in Immunosuppressed Mice

**Figure 3:**
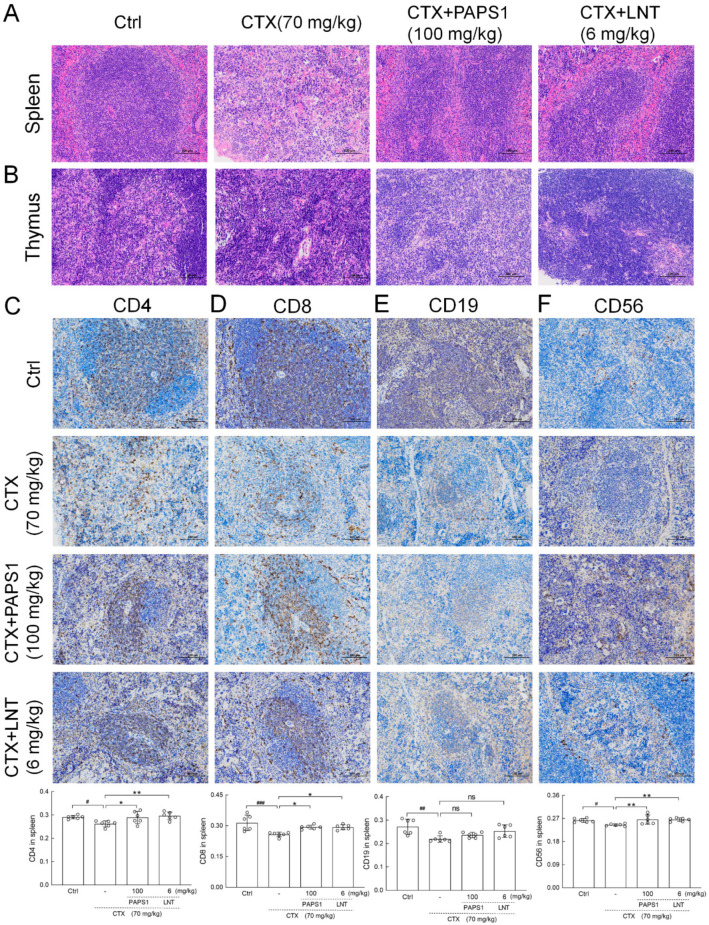
PAPS1 improved the histopathological damage caused by CTX in the spleen and thymus, and it increased the immunophenotype expression of immune cells in the spleen. Histopathological observation of the (**A**) spleen and (**B**) thymus (magnification: 200×, scale bar: 100 μm). Immunohistochemical staining of (**C**) CD4, (**D**) CD8, (**E**) CD19 and (**F**) CD56 in the spleens of mice (magnification: 200×, scale bar: 100 μm). The average optical density is represented on the y-axis. Data are presented as the mean ± S.D. (*n* = 6) and were analyzed via a one-way ANOVA test followed by Dunn’s multiple comparisons post hoc tests. ^#^ *p* < 0.05, ^##^ *p* < 0.01, ^###^ *p* < 0.001, compared with the Ctrl mice; ns > 0.05, * *p* < 0.05, ** *p* < 0.01, compared with CTX-only treated mice. The height of the column chart represents the means, and the circle represents the independent sample value within each group. Ctrl, control, CTX, cyclophosphamide; PAPS1, water-soluble PA polysaccharides, LNT, lentinan.

Compared with control mice, the body weight and thymus index of CTX-only treated mice were drastically decreased (*p* < 0.001) (Table S4), and no significant changes were noted after the PAPS1 and LNT treatments (Table S4). H&E staining revealed a disrupted splenic architecture in the immunosuppressed mice and an increased number of the multinucleated giant cells compared with the control (Figure 3A). A loss of architectural organization was also observed in the thymus of CTX-treated mice, together with a decrease in lymphatic cells and an increase in interstitial tissue, compared with the control group (Figure 3B). All CTX-induced changes were reversed by PAPS1 and LNT administration. PAPS1 prevented any histopathological changes in the liver and the kidney of CTX-injected mice (Figure S3).

In the spleen, CTX injection reduced the expressions of CD4 (*p* < 0.05) (Figure 3C) and CD8 (*p* < 0.001) (Figure 3D), compared with the control group. These CTX-induced effects were reversed by PAPS1 (*p* < 0.05) (Figure 3C,D) and LNT (*p* < 0.05) (Figure 3C,D). Furthermore, PAPS1 resulted in 13.05% and 13.67% increments in the expression of CD4 (*p* < 0.05) (Figure 3C) and CD8 (*p* < 0.05) (Figure 3D) in the spleens of immunosuppressed mice. CTX injection reduced the expression of CD19 (*p* < 0.01) (Figure 3E) and CD56 (*p* < 0.05) (Figure 3F) in the spleens, whereas PAPS1 enhanced 8.50% of the expression of CD56 (*p* < 0.01) (Figure 3F) without influencing the level of CD19 (*p* > 0.05) (Figure 3E). 

**Figure 4:**
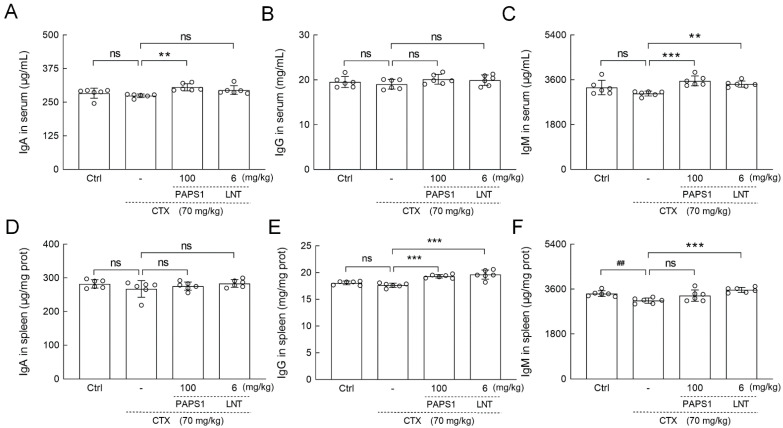
PAPS1 increased the levels of Igs in the spleen and the serum of CTX-immunosuppressed mice. The levels of (**A**) IgA, (**B**) IgG and (**C**) IgM in the serums and (**D**) IgA, (**E**) IgG and (**F**) IgM in the spleens of mice were analyzed by ELISA. The data are presented as the mean ± S.D. (*n* = 6) and were analyzed via a one-way ANOVA test followed by Dunn’s multiple comparisons post-hoc tests. ns > 0.05, ^##^ *p* < 0.01 compared with Ctrl mice; ns > 0.05, ** *p* < 0.01, *** *p* < 0.001 compared with CTX-treated mice. The height of the column chart represents the means, and the circle represents the independent sample value within each group. Ctrl, control, CTX, cyclophosphamide; PAPS1, water-soluble PA polysaccharides, LNT, lentinan. Ig, immunoglobulin; ELISA, enzyme-linked immunosorbent assay.

Compared with CTX-only treated mice, PAPS1 resulted in increments in immunoglobulin (IgA) (11.53%) (*p* < 0.01) (Figure 4A) and IgM (16.61%) (*p* < 0.001) (Figure 4C) in serum levels and increments in the splenic level of IgG (9.50%) (*p* < 0.001) (Figure 4E). LNT showed immunoregulatory effects similar to those of PAPS1 on the expressions of CD4, CD8, CD19, CD56 and Igs levels (Figure 3 and Figure 4). 

### 3.4. PAPS1 Regulated Intestinal Microflora in Immunosuppressed Mice

**Figure 5:**
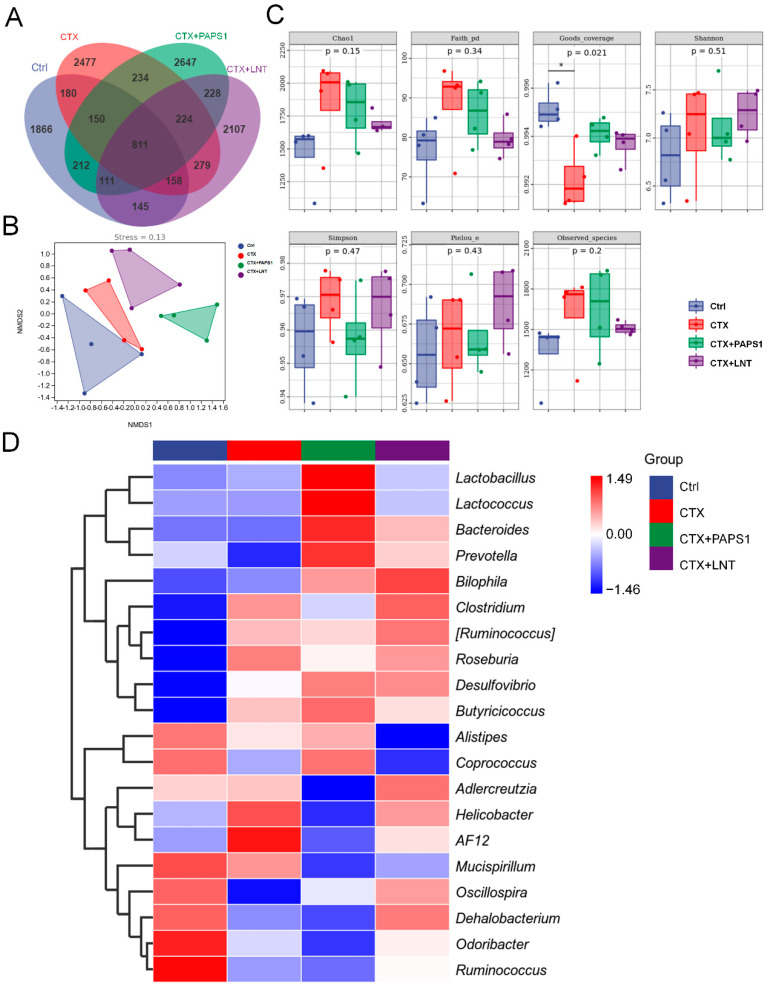
PAPS1 regulated the intestinal microflora of CTX-immunosuppressed mice: (**A**) Venn diagram. (**B**) NMDS of unweighted UniFrac distance from beta diversity analysis. (**C**) Chao1, Faith’s phylogenetic diversity (Faith pd), Good’s coverage, Shannon’s and Simpson’s indices, Pielou’s evenness and the observed species index values from alpha diversity analysis. Data were analyzed using a one-way ANOVA and are expressed as mean ± S.D. (*n* = 4). * *p* < 0.05 compared with Ctrl mice. (**D**) Heatmap of 20 bacterial genera with the most significantly different abundance, calculated from the unweighted UniFrac distance of cecal content samples. NMDS, non-metric multi-dimensional scaling. Ctrl, control, CTX, cyclophosphamide; PAPS1, water-soluble PA polysaccharides, LNT, lentinan.

The 16S rRNA gene sequences were clustered into operational taxonomic units (OTUs), using a clustering threshold of 97%. Compared with CTX-only treated mice, the number of specific OTUs in PAPS1-treated mice was 1419 (31.4% of the number in CTX-only treated mice) (Figure 5A), indicating relatively large differences in the intestinal microflora composition among the groups. The microbial composition between the CTX and PAPS1-treated mice showed a specific separation (Figure 5B). However, compared with CTX, both PAPS1 and LNT failed to influence alpha diversity (Figure 5C). Based on beta diversity, a heatmap of the top 20 bacterial genera presents the most significantly different abundances, as presented in Table 3 and Figure 5D (detailed information shown in Tables S5 and S6). The abundances of four bacterial genera (*Prevotella*, *Alistipes*, *Coprococcus* and *Oscillospira*) were decreased by CTX injection and were increased by PAPS1. Furthermore, PAPS1 reversed the increase in the abundance of *Clostridium*, *Roseburia*, *Helicobacter* and *AF12* caused by the CTX injection (Figure 5D). Compared to the control mice, one of the most specific dominant nodes of intestinal microflora was *Helicobacter* (*p* < 0.05) (Table 3) in CTX-only treated mice and *Bacteroidales* in PAPS1-treated mice (*p* < 0.05) (Table 3). This difference had an impact on immunity and oxidative stress. 

### 3.5. PAPS1 Regulated Cytokines in Immunosuppressed Mice

**Figure 6:**
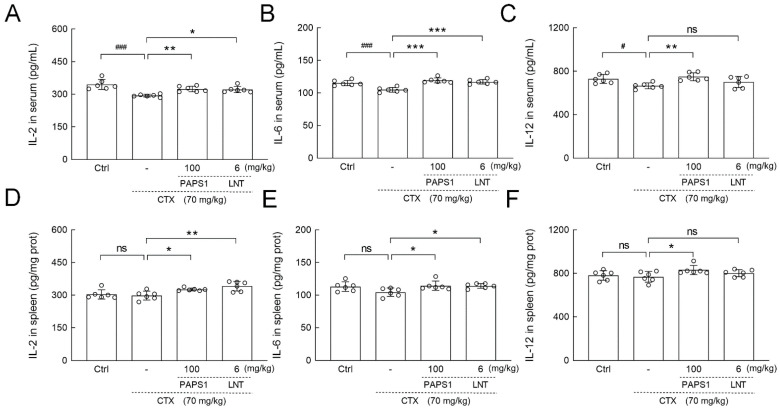
PAPS1 regulated the levels of inflammatory cytokines in the serums and spleens of CTX-immunosuppressed mice. The levels of (**A**) IL-2, (**B**) IL-6, (**C**) IL-12 in the serums, and (**D**) IL-2, (**E**) IL-6, (**F**) IL-12 in the spleens were detected by ELISA. Data are presented as the mean ± S.D. (*n* = 6) and were analyzed via a one-way ANOVA test followed by Dunn’s multiple comparisons post hoc tests. ns > 0.05, ^#^ *p* < 0.05 and ^###^ *p* < 0.001, compared with Ctrl mice; ns > 0.05, * *p* < 0.05, ** *p* < 0.01 and *** *p* < 0.001, compared with CTX-only treated mice. The height of the column chart represents the means, and the circle represents the independent sample value within each group. Ctrl, control, CTX, cyclophosphamide; PAPS1, water-soluble PA polysaccharides, LNT, lentinan; IL, Interleukin.

Compared with the control, CTX injections reduced the serum levels of IL-2 (14.84%) (*p* < 0.001) (Figure 6A), IL-6 (9.01%) (*p* < 0.001) (Figure 6B) and IL-12 (8.73%) (*p* < 0.05) (Figure 6C), whereas PAPS1 increased their serum levels by 10.31%, 13.67% and 12.58% (*p* < 0.01) (Figure 6), respectively. Compared to CTX, PAPS1 administration significantly increased the splenic levels of IL-2 (9.39%) (*p* < 0.05) (Figure 6D), IL-6 (9.57%) (*p* < 0.05) (Figure 6E) and IL-12 (8.08%) (*p* < 0.05) (Figure 6F), respectively. LNT showed similar effects to PAPS1 regarding the regulation of IL-2 (*p* < 0.05) (Figure 6A) and IL-6 (*p* < 0.001) (Figure 6B) in the serums and IL-2 (*p* < 0.01) (Figure 6D) and IL-6 (*p* < 0.05) (Figure 6E) in the spleens of immunosuppressed mice. 

### 3.6. PAPS1 Suppressed Oxidative Stress in Immunosuppressed Mice

**Figure 7:**
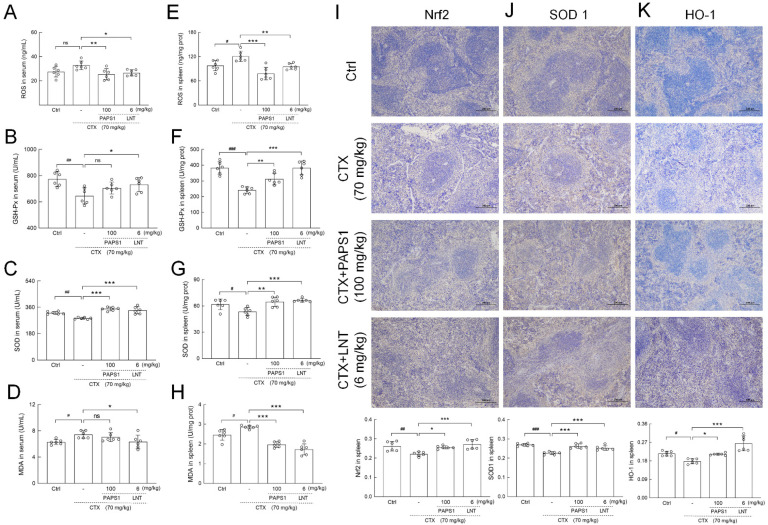
PAPS1 regulated the levels of oxidative stress indices and expressions of Nrf2 and its downstream proteins in the serums and spleens of CTX-immunosuppressed mice. The levels of (**A**) ROS, (**B**) GSH-Px, (**C**) SOD and (**D**) MDA in the serums and the levels of (**E**) ROS, (**F**) GSH-Px, (**G**) SOD and (**H**) MDA in the spleens were measured. Immunohistochemical staining of (**I**) Nrf2, (**J**) SOD1 and (**K**) HO-1 in the spleens of mice (magnification: 100×, scale bar: 200 μm). Data are presented as the mean ± S.D. (*n* = 6) and were analyzed via a one-way ANOVA test followed by Dunn’s multiple comparisons post hoc tests. ns > 0.05, ^#^ *p* < 0.05, ^##^ *p* < 0.01 and ^###^ *p* < 0.001, compared with Ctrl mice; ns > 0.05, * *p* < 0.05, ** *p* < 0.01 and *** *p* < 0.001, compared with CTX-only treated mice. The height of the column chart represents the means, and the circle represents the independent sample value within each group. Ctrl, control, CTX, cyclophosphamide; PAPS1, water-soluble PA polysaccharides, LNT, lentinan; Nrf2, nuclear factor erythroid 2-related factor 2; ROS, reactive oxygen species; GSH-Px, glutathione peroxidase; SOD, superoxide dismutase; MDA, malondialdehyde; SOD1, superoxide dismutase 1; HO-1, heme oxygenase 1.

Compared with control mice, CTX had increased serum and splenic reactive oxygen species (ROS) levels by 19.46% (*p* > 0.05) (Figure 7A) and 23.63% (*p* < 0.05) (Figure 6E). PAPS1 reduced serum and splenic ROS levels by 22.82% (*p* < 0.01) (Figure 7A) and 35.40% (*p* < 0.001) (Figure 7E), respectively, compared with CTX administration. Compared to CTX, PAPS1 reversed the reductions in superoxide dismutase (SOD) by 22.42% (*p* < 0.001) (Figure 7C) in the serums and 21.05% (*p* < 0.01) (Figure 7G) in the spleens, enhanced the level of glutathione peroxidase (GSH-Px) by 29.41% (*p* < 0.01, Figure 7F) in the spleen, and it suppressed the level of malondialdehyde (MDA) by 30.95% in the spleens (*p* < 0.01) (Figure 7H). Similar with PAPS1, compared to CTX, LNT regulated the levels of ROS, GSH-Px, SOD and MDA in the serums and spleens of immunosuppressed mice (*p* < 0.05) (Figure 7A–H). Nrf2 acts as a cytoprotective factor by regulating the expression of down-stream antioxidant genes, including heme oxygenase 1 (HO-1) and SODs. Compared with NS, CTX caused reductions in the expressions of Nrf2 (*p* < 0.01) (Figure 7I), HO-1 (*p* < 0.001) (Figure 7J) and superoxide dismutase 1 (SOD1) (*p* < 0.05) (Figure 7K). This was reversed by PAPS1 (*p* < 0.05) and LNT (Figure 7).

## 4. Discussion

In this study, PAPS1, with an Mw of 17.16 kDa, was isolated and purified from the fruiting bodies of *P. abieticola*. PAPS1 has a main backbone containing →6)-α-D-Gal*p*-(1→, →2,6)-α-D-Gal*p*-(1→ and →3)-β-D-Glc*p*-(1→ residues. Branches mainly include β-D-Man*p*-(1→ and β-D-Man*p*-(1→6)-α-D-Gal*p* -(1→, which are attached at the C-2 of the →2,6)-α-D-Gal*p*-(1→ residue. Polysaccharide fragments containing β-1,3/1,6-glucans have been shown to possess immunomodulatory properties [30]. In CTX-immunosuppressed mice, PAPS1 reversed reductions in NK cells and T cells and regulated the levels of immunoglobulins and other immunological factors showing the immunity-enhancing effect. 

Immunosuppression is a temporary or long-term immune dysfunction. CTX, one of the most widely used immunosuppressants, is wildly used for cancer therapy. In this work, CTX caused histopathological damage in the spleen and thymus and reduced the activity of T cells, B cells and NK cells. High dosages of CTX reduce the number of dendritic cells (DCs) and alter the polarization of Th cells, inducing immunosuppression [31]. The DCs of CD8^+^ T and CD56^+^ NK cells are activated, enhancing the cross-activation of adaptive and innate immune responses [32]. Polysaccharides from *Pleurotus eryngii* with a branch of β-1,6-glucan increase the proportion of CD4 T and CD8 T cells to improve immunity [33]. According to our results, PAPS1 not only enhanced the splenic expressions of CD4, CD8 and CD56 in immunosuppressed mice, but it also increased the IgA and IgM serum levels. IgM, which mostly exists in the serum, enhances antigen presentation and the downstream immune response [34]. Moreover, IgA enhances the diversity of intestinal microflora and contributes to the elimination of pathogens by combining with the IgA Fc receptor (FcαRI; CD89) to activate immune cells [35]. Taken together, these data suggest that PAPS1 enhances immune functions in mice. 

It has been reported that *Ganoderma lucidum* polysaccharides enhance Th1 responses with high levels of IL-2 [36]. PAPS1 regulates serum and splenic ILs levels in immunosuppressed mice, preventing decreases in CTX-induced IL-6 and IL-12. IL-12, produced by DCs, synergizes with IL-2, produced by CD4^+^ T cells, and increases the activity of NK cells to release lytic molecules such as interferon γ [37]. IL-6 secretion is directly induced by TNF, and it promotes the production of Igs [38]. Furthermore, IL-2 enhances the activation of CD4^+^ and CD8^+^ effector T cells and, consequently, enhances the immunity as well [39]. PAPS1 enhanced the immune function related to its regulation of NK and T cells, further leading to the release of ILs. 

Immune dysfunction leads to an imbalance in intestinal microflora, which in turn causes more serious immune disorders [40]. An increasing number of studies has shown that polysaccharides from medical mushrooms regulate immunity and gut microbiota in mice with CTX. A polysaccharide from *Cordyceps sinensis* has been reported to possibly enhance intestinal immunity and regulate the balance of T helper (Th)1/Th2 cells [41]. *Lycium barbarum* polysaccharides not only restore damage-immune organs and adjust T cells, but also they also increase the abundances of *Prevotellaceae*, which is positively associated with immune regulation [42]. In this study, the dominant bacterial species of the CTX-immunosuppressed mice intestinal microflora belonged to the Proteobacteria phylum. *Helicobacter pylori* belongs to this group. It inhibits the maturation of DC and reduces antigen processing, decreasing the maturation and response of T cells [43,44]. The dominant bacterial species in the intestinal microflora of PAPS1-treated mice mainly belongs to the Bacteroidete phylum, similar to the control (healthy) mice. Bacteroidetes members can digest polysaccharides into short chains, improving the immune response of the host [45]. *Bacteroides ovatus* regulates the T-cell-dependent IgA response [46]. Additionally, *Bacteroides* can promote the development and maturation of IgA-secreting cells, further enhancing the immune function of the host [47].

Accordingly, bacteria from the Bacteroidales order decrease oxidative stress by reducing ROS and MDA levels [48]. The toxin secreted by enterotoxigenic *Bacteroides fragilis* stimulates DCs, resulting in the upregulation of HO-1 in C57BL/6 mice [49]. In immunosuppressed mice, PAPS1 prevented the increase in ROS and MDA levels and enhanced the expression levels of Nrf2 and its downstream proteins. ROS such as H_2_O_2_ induce apoptosis in both NK and T cells, leading to immune suppression. Nrf2 can prevent the damage of T and NK cells by reducing the level of ROS [50]. 

In this study, we confirmed that changes in the intestinal microflora composition caused by PAPS1 lead to an increase in the concentration of IL, thus stimulating the activity of T and NK cells and enhancing immunity, protecting from the side effects of CTX treatment. A limitation of this study is that we failed to investigate the structure–function relationship for PAPS1. A higher replication of the sample should be used in further studies to investigate the mechanism of PAPS1 regarding intestinal microflora regulation.

## 5. Conclusions

In conclusion, we demonstrated that a water-soluble polysaccharide component isolated from *P. abieticola* possesses immune enhancement activity by promoting the immune cell response, and the mechanism underlying the immune enhancement might involve its regulation of intestinal-microbiome-mediated oxidative stress. Our data suggest that PAPS1 is expected to be a potential relieving agent or functional food to alleviate the immunosuppressive side effects of CTX. Further studies should be performed to develop treatments based on the functions of PAPS1 and other natural ingredients. 

## Figures and Tables

**Table 1:** Methylation analysis of PAPS1.

Retention Time (min)	Linkage Pattern	Methylated Sugar	Characteristic Ions (*m*/*z*)	Relative Mole Percentage * (%)
6.773	*t*-Fuc(*p*)	1,5-di-O-acetyl-6-deoxy-2,3,4-tri-O-methyl fucitol	59, 72, 89, 102, 115, 118, 131, 162, 175	1.81
8.624	*t*-Man(*p*)	1,5-di-O-acetyl-2,3,4,6-tetra-O-methyl mannitol	59, 71, 87, 102, 113, 118, 129, 145, 161, 162, 205, 246	32.19
9.683	*t*-Gal(*p*)	1,5-di-O-acetyl-2,3,4,6-tetra-O-methyl galactitol	59, 71, 87, 102, 113, 118, 129, 145, 161, 162, 205	1.67
11.908	3-Glc(*p*)	1,3,5-tri-O-acetyl-2,4,6-tri-O-methyl glucitol	59, 71, 74, 87, 101, 118, 129, 143, 161, 174, 203, 217, 234, 277	2.82
15.228	6-Gal(*p*)	1,5,6-tri-O-acetyl-2,3,4-tri-O-methyl galactitol	59, 71, 87, 99, 102, 118, 129, 143, 159, 162, 189, 204, 233	31.59
19.398	2,6-Gal(*p*)	1,2,5,6-tetra-O-acetyl-3,4-di-O-methyl galactitol	60, 74, 87,100, 114, 130, 143, 160, 174, 190, 204, 234	29.93

* Relative mole percentage * (%) = Relative molar mass/Sum of the relative molar mass of components.

**Table 2:** ^1^H and ^13^C NMR chemical shifts for PAPS1.

Residue	Glycosyl Residues	H1/C1	H2/C2	H3/C3	H4/C4	H5/C5	H6a /C6	H6b	Reference
A	β-D-Man*p*-(1→	4.72	4.08	3.57	3.75	3.48	3.85	3.66	[16]
		101.69	72.93	76.25	70.43	75.67	61.07		
B	→6)-α-D-Gal*p*-(1→	4.90	3.78	4.01	4.08	4.21	3.85	3.50	[26]
		98.30	69.19	69.50	69.63	68.54	66.82		
C	→2,6)-α-D-Gal*p*-(1→	5.04	3.82	3.91	4.00	4.10	3.85	3.58	[27]
		98.15	76.97	69.20	70.43	68.81	66.78		
D	→ 3)-β-D-Glc*p*-(1→	4.47	3.64	4.25	3.51	3.48	3.29	3.22	[28,29]
		102.08	73.17	78.80	70.50	75.65	60.26		

**Table 3:** Dominant nodes of intestinal microflora in mice.

Group	Taxa	Abundance (log10)	LDA Score	*p*
Ctrl	Bacteria.Firmicutes.Erysipelotrichi.Erysipelotrichales	4.185	3.362	0.022
	Bacteria.Firmicutes.Clostridia.Clostridiales.Ruminococcaceae.*Ruminococcus*	4.185	3.697	0.019
	Bacteria.Firmicutes.Bacilli.Lactobacillales.Streptococcaceae.*Streptococcus*	4.158	2.534	0.032
	Bacteria.Firmicutes.Erysipelotrichi.Erysipelotrichales.Erysipelotrichaceae	3.714	3.379	0.022
	Bacteria.Bacteroidetes.Bacteroidia.Bacteroidales._Odoribacteraceae_	3.714	3.653	0.02
	Bacteria.Proteobacteria.Gammaproteobacteria.Pseudomonadales.Moraxellaceae.*Perlucidibaca*	3.714	2.41	0.017
	Bacteria.Firmicutes.Erysipelotrichi	2.91	3.365	0.022
	Bacteria.Bacteroidetes.Bacteroidia.Bacteroidales._Odoribacteraceae_.*Odoribacter*	1.952	3.623	0.02
Model	Bacteria.Proteobacteria.Epsilonproteobacteria	4.19	3.835	0.029
	Bacteria.Proteobacteria.Epsilonproteobacteria.Campylobacterales.Helicobacteraceae.*Helicobacter*	4.19	3.838	0.029
	Bacteria.Proteobacteria.Epsilonproteobacteria.Campylobacterales.Helicobacteraceae	4.19	3.787	0.029
	Bacteria.Chloroflexi.Anaerolineae.SBR1031	4.19	2.738	0.009
	Bacteria.Chloroflexi.Anaerolineae.SBR1031.A4b	2.078	3.3	0.017
	Bacteria.Proteobacteria.Epsilonproteobacteria.Campylobacterales	2.03	3.836	0.029
	Bacteria.Chloroflexi.Anaerolineae.SBR1031.SHA_31	1.911	2.44	0.007
	Bacteria.Chloroflexi.Anaerolineae	1.409	2.538	0.03
PAPS1	Bacteria.Bacteroidetes.Bacteroidia.Bacteroidales._Paraprevotellaceae_.*Paraprevotella*	2.711	2.461	0.045
	Bacteria.Bacteroidetes.Bacteroidia.Bacteroidales._Paraprevotellaceae_	2.711	2.44	0.039
	Bacteria.Proteobacteria.Gammaproteobacteria.Alteromonadales.Idiomarinaceae	1.72	2.774	0.017
LEP	Bacteria.Firmicutes.Clostridia.Clostridiales.Lachnospiraceae.*Clostridium*	3.201	2.704	0.014
	Bacteria.Firmicutes.Clostridia.Clostridiales.Ruminococcaceae.*Ruminococcus.Ruminococcus_albus*	1.545	2.95	0.017

Data are presented as the mean (*n* = 4) and were analyzed using a one-way ANOVA test followed by Dunn’s multiple comparisons post hoc test. The sizes of dominant nodes were proportional to the relative abundance of the operational taxonomic units (measured as log 10 of median reads). The LDA score and *p* value were calculated. Ctrl, control, CTX, cyclophosphamide; PAPS1, water-soluble PA polysaccharides, LNT, lentinan.

## Data Availability

Not applicable.

## Supplementary Materials

The following supporting information can be downloaded at: https://www.mdpi.com/article/10.3390/nu14204410/s1, Figure S1: Methylation GC–MS chart of PAPS1; Figure S2: Structural characterization of PAPS1. Two-dimensional NMR atlas of (A) 1H-1H COSY, (B) HSQC, (C) 1H-1H NOESY and (D) HMBC; Figure S3: PAPS1 showed no histopathological effect on the livers and kidneys of CTX-injected mice. H&E staining and histopathological observation of the (A) liver and (B) kidney (magnification: 200×, scale bar: 100 μm); Table S1: Primary antibodies used for immunohistochemistry staining; Table S2: ELISA kits used for the assessment of biochemical indices; Table S3: Molecular characteristics of PAPS1; Table S4: Effects of PAPS1 on body weight and organ index in 70 mg/kg-CTX-induced immunosuppressed mice; Table S5: The number of microbial taxa at different levels of intestinal microflora in mice; Table S6: The relative abundance of each node of intestinal microflora in mice. References [20,22] are cited in the supplementary materials.
