# Supplementary material for: The Structural Characterization and Immunomodulatory Activity of Polysaccharides from Pleurotus abieticola Fruiting Bodies
Source: Nutrients. 2022 Oct 20;14(20):4410. doi: 10.3390/nu14204410 (PMC9607439; doi:10.3390/nu14204410)
Supplement: Supplementary file 1 [file nutrients-14-04410-s001.zip › nutrients-1917784-supplementary.pdf]

Supplementary Information

Methods for methylation and NMR analysis

1. Experimental conditions for methylation

As previously described [1], the bonding structure of PAPS1 was determined. Briefly, after derivatization included methylation and acetylation, PAPS1 was analysed by gas chromatography-mass spectrometer (GC-MS) (7890A GC coupled with 5977B inert XL MS detector; Agilent Technologies Inc. CA, UAS). According to the properties of compounds, the injection volume was 1 μL, the split ratio was 10:1, the carrier gas was high-purity helium. The oven temperature was programmed from 140℃ (2.0 min) to 230℃ (3 min) at a ramp of 3℃. The quadrupole mass spectrometry detection system (Agilent 5977B; Agilent Technologies, USA) was adopted for the mass spectrometry system. The mass spectrometry was equipped with an electron impact ion source (EI) and a MassHunter workstation. The detection was under a full scan mode, with a mass scan range of 30-600 m/z.

2. **Experimental conditions for NMR**

Following the method previously published by our group [2], after the sample (15mg) dissolved in 500 uL D_2O, and the sample solution was filtered and transferred to a NMR tube. ^{1}H, ^{13}C, ^{1}H-^{1}H correlation spectroscopy (COSY), heteronuclear single quantum correlation (HSQC), heteronuclear multiple bond correlation (HMBC) and nuclear overhauser effect spectroscopy (NOESY) were recorded using Bruker Avance AV600 NMR spectrometer (500 MHz for ^{1}H, 128 MHz for ^{13}C; Bruker, Germany). NMR spectrometer were equipped with liquid QXI $^{1}H/^{31}P/^{13}C/^{15}N$ quadrupole resonance probe (Z-gradient; 5mm; Bruker, Germany). The technical parameters were as follows: signal to noise ratio (^{1}H), 888; resolution (Hz), 0.32 (rotating). The technical parameters of liquid QXI $^{1}H/^{31}P/^{13}C/^{15}N$ quadrupole resonance probe (Z-gradient) were as follows: signal to noise ratio (^{1}H), 798; resolution (Hz), 0.26 (rotating), and signal to noise ratio (^{13}C), 328; resolution (Hz), 0.1. NMR data were processed using MestReNova-14.0.0-23239 (Mestrelab Research, Santiago de Compostela, Spain).

References for "Methods for methylation and NMR analysis"

[1] W. Hu, M. Song, C. Wang, Z. Guo, Y. Li, D. Wang, Structural characterization of polysaccharide purified from Hericium erinaceus fermented mycelium and its pharmacological basis for application in Alzheimer's disease: Oxidative stress related calcium homeostasis, International journal of biological macromolecules (2021) 193(Pt A), 358-369.
[2] J. Wang, J. Song, D. Wang, N. Zhang, J. Lu, Q. Meng, Y. Zhou, N. Wang, Y. Liu, D. Wang, L. Teng, The anti-membranous glomerulonephritic activity of purified polysaccharides from Irpex lacteus Fr, International journal of biological macromolecules (2016) 84, 87-93.

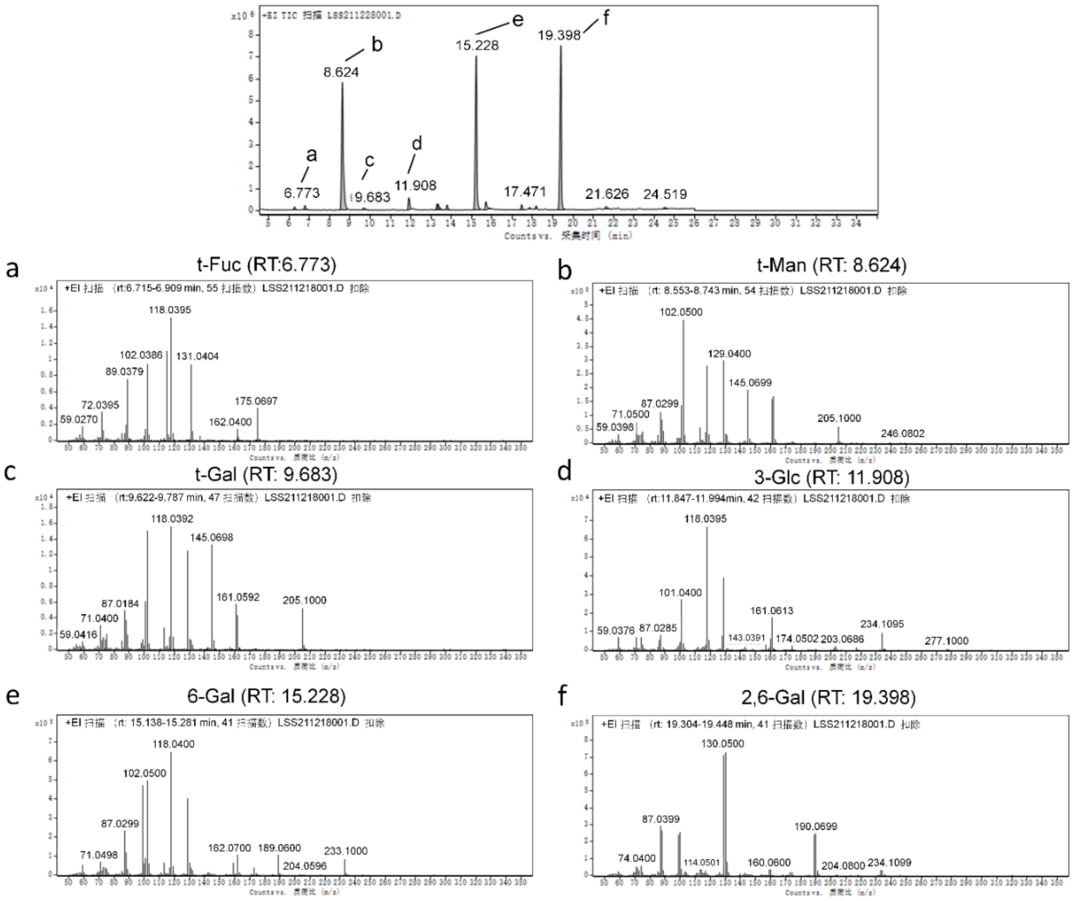

Figure S1. Methylation GC-MS chart of PAPS1. GC-MS, gas chromatography–mass spectrometry; PAPS1, water-soluble PA polysaccharides; Fuc, fucose; Man, mannose; Gal, galactose; Glc, glucose; RT, retention time.

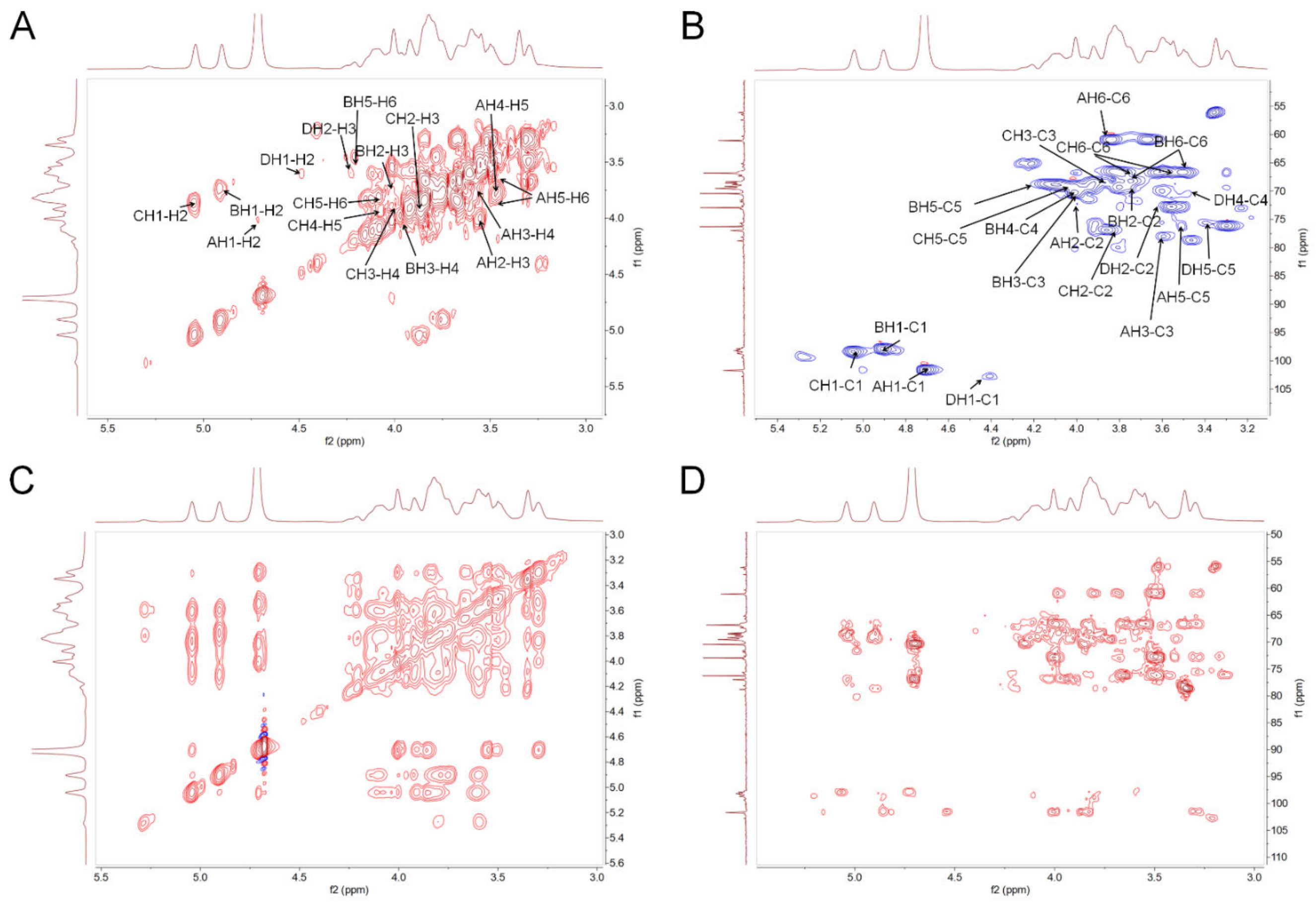

Figure S2. Structural characterization of PAPS1. Two-dimensional NMR atlas of (A) ^{1}H-^{1}H COSY, (B) HSQC, (C) ^{1}H-^{1}H NOESY and (D) HMBC. PAPS1, water-soluble PA polysaccharides; NMR, Nuclear magnetic resonance; COSY, correlation spectroscopy; HSQC, heteronuclear single quantum correlation; NOESY, nuclear overhauser effect spectroscopy; HMBC, heteronuclear multiple bond cor-relation.

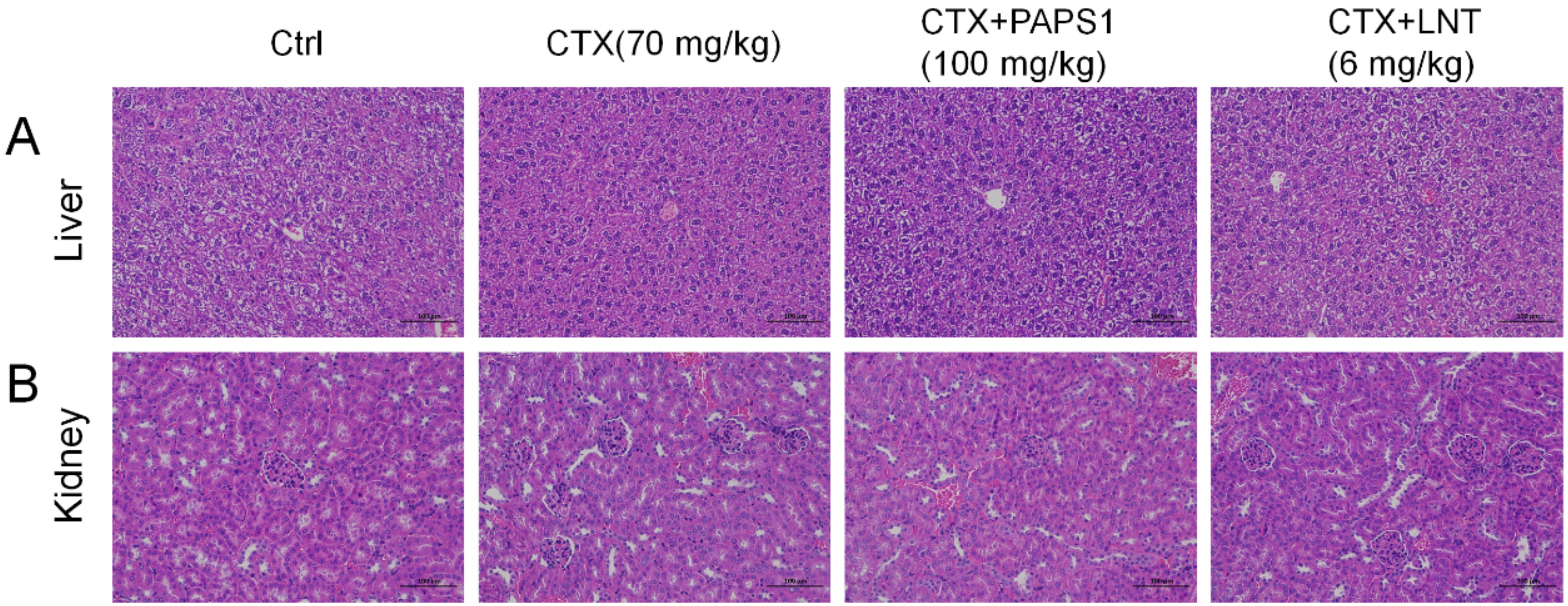

Figure S3. PAPS1 showed no histopathological effect on liver and kidney of CTX-injected mice. H&E staining and histopathological observation of (**A**) liver and (**B**) kidney (magnification: 200×, scale bar: 100 μm). PAPS1, water-soluble PA polysaccharides; CTX; cyclophosphamide.

Table S1. Primary antibodies used for immunohistochemistry staining.

Name	Item number	Dilution rate	Manufacturer*
CD4	GB11064	1:500	Wuhan Service
CD8	GB13429	1:500	Wuhan Service
CD19	GB11061-1	1:500	Wuhan Service
CD56	GB112671	1:500	Wuhan Service
Nrf2	GB113808	1:500	Wuhan Service
HO-1	GB11104	1:500	Wuhan Service
SOD1	10269-1-AP	1:500	Proteintech Group

*Primary antibodies were all purchased from Wuhan Service Biological Co., Ltd (Wuhan, Hubei Province, China) and Proteintech Group Inc (Wuhan, China). Nrf2, nuclear factor erythroid 2-related factor 2; SOD1, superoxide dis-mutase 1; HO-1, heme oxygenase 1.

Table S2. ELISA kits used for biochemical indices assessment.

Name	Item number	Manufacturer
IL-2	ml063136-J	Shanghai Enzyme-linked Biotechnology Co., Ltd
IL-6	ml002293-J	Shanghai Enzyme-linked Biotechnology Co., Ltd
IL-12	ml037868	Shanghai Enzyme-linked Biotechnology Co., Ltd
Ig A	ml037606	Shanghai Enzyme-linked Biotechnology Co., Ltd
IgG	ml037601	Shanghai Enzyme-linked Biotechnology Co., Ltd
IgM	ml063597	Shanghai Enzyme-linked Biotechnology Co., Ltd
ROS	ml009876-1	Shanghai Enzyme-linked Biotechnology Co., Ltd
SOD	A001-3-2	Nanjing Jiancheng Bioengineering Institute
GSH-Px	A005-1-2	Nanjing Jiancheng Bioengineering Institute
MDA	A003-1-1	Nanjing Jiancheng Bioengineering Institute

IL, interleukin; Ig, immunoglobulin; ROS, reactive oxygen species; GSH-Px, glutathione peroxidase; SOD, superoxide dismutase; MDA, malondialdehyde.

Table S3. Molecular characteristics of PAPS1.

**Polydispersity**	
Mw/Mn	1.104
Mz/Mn	1.422
**Molar mass moments (10^4g/mol)**	
Mn	1.555
Mp	1.478
Mw	1.716
Mz	2.211

Note: Mn: number-average molecular weight; Mp: peak molecular weight; Mw: weight-average molecular weight; Mz: Z-average molecular weight.

Table S4. Effects of PAPS1 on body weight and organ index in 70 mg/kg CTX induced immunosuppressive mice.

		Ctrl	CTX （70mg/kg）		
			Model	LNT （6 mg/kg）	PAPS1 （100 mg/kg）
Body weight (g)	0	22.5±0.4	22.6±0.5	22.4±0.4	22.3±0.5
	7th	23.6±0.3	20.8±0.3###	20.9±0.6	20.8±0.4
	14th	24.1±0.4	21.2±0.6###	21.7±0.3*	21.4±0.4
	21st	24.7±0.4	21.9±0.6###	22.4±0.6	22.1±0.7
	28th	25.5±0.5	22.2±0.4###	23.1±0.5**	22.4±0.8
	35th	26.0±0.7	22.7±0.5###	23.6±0.6**	22.9±0.8
Organ index (mg/g)	Spleen	3.6±0.2	3.5±0.4	3.4±0.2	4.1±0.4***
	Thymus	1.5±0.3	0.7±0.1###	0.7±0.1	0.8±0.2

Data are present as the mean ± S.D. (n = 10) and analyzed via a one-way ANOVA test followed by post-hoc Dunn's multiple comparison tests. $^{##}p < 0.01$, $^{###}p < 0.001$, compared with Ctrl group; $^{*}p < 0.05$, $^{**}p < 0.01$, $^{***}p < 0.001$, compared with Model group. Ctrl, control, CTX, cyclophosphamide; PAPS1, water-soluble PA polysaccharides, LNT, lentinan.

Table S5. The number of microbial taxa at different levels of intestinal microflora in mice.

Group	phylum	class	order	family	genus	species
Ctrl	11	18	24	34	37	15
	10	17	24	33	40	13
	10	20	29	39	42	14
	10	19	25	38	37	15
Model	11	18	26	39	39	19
	11	20	24	40	43	21
	11	18	22	36	37	21
	10	17	21	33	31	13
PAPS1	11	19	23	42	44	22
	11	17	23	37	39	23
	9	17	23	43	50	26
	9	16	25	41	47	20
LNT	9	16	24	41	44	18
	8	15	20	34	39	21
	9	15	19	35	44	18
	11	18	21	32	34	16

Ctrl, control, CTX, cyclophosphamide; PAPS1, water-soluble PA polysaccharides, LNT, lentinan.

Table S6. The relative abundance of each node of intestinal microflora in mice.

	Taxa	Abundace（log10）	LDA score	Group
1	Bacteria.Firmicutes.Bacilli.Lactobacillales.Streptococcaceae	3.597		
2	Bacteria.Firmicutes.Clostridia.Clostridiales.Eubacteriaceae	1.270		
3	Bacteria.Actinobacteria.Actinobacteria.Actinomycetales.Micromonosporaceae	1.099		
4	Bacteria.Proteobacteria.Alphaproteobacteria.Rhizobiales.Brucellaceae.Ochrobactrum	0.950		
5	Bacteria.Firmicutes.Clostridia.Clostridiales.Clostridiaceae.Candidatus_Arthromitus	2.174		
6	Bacteria.Proteobacteria.Betaproteobacteria.Burkholderiales.Comamonadaceae.Curvibacter	1.596		
7	Bacteria.Actinobacteria.Actinobacteria.Actinomycetales.Micrococcaceae.Arthrobacter	1.792		
8	Bacteria.Proteobacteria.Gammaproteobacteria.Xanthomonadales.Xanthomonadaceae.Stenotrophomonas.Stenotrophomonas_geniculata	1.196		
9	Bacteria.Acidobacteria.Sva0725	0.880		
10	Bacteria.Firmicutes.Clostridia.Clostridiales.Dehalobacteriaceae	3.779		
11	Bacteria.Proteobacteria.Betaproteobacteria.Rhodocyclales.Rhodocyclaceae.Azoarcus	1.424		
12	Bacteria.Acidobacteria._Chloracidobacteria_	1.204		
13	Bacteria.Proteobacteria.Alphaproteobacteria.Rhizobiales.Phyllobacteriaceae	0.943		
14	Bacteria.Firmicutes.Bacilli.Bacillales.Bacillaceae.Bacillus	1.686		
15	Bacteria.Firmicutes.Clostridia.Clostridiales.Dehalobacteriaceae.Dehalobacterium	3.779		
16	Bacteria.Bacteroidetes	5.706		
17	Bacteria.Bacteroidetes.Bacteroidia.Bacteroidales.Porphyromonadaceae.Macellibacteroides.Macellibacteroides_fermentans	0.798		
18	Bacteria.Firmicutes.Bacilli.Bacillales.Planococcaceae	1.826		
19	Bacteria.Chloroflexi.Anaerolineae.Ardenscatenales.Ardenscatenaceae.Ardenscatena	1.099		
20	Bacteria.Firmicutes.Bacilli.Lactobacillales.Lactobacillaceae.Lactobacillus.Lactobacillus_iners	1.196		
21	Bacteria.Firmicutes.Clostridia.Clostridiales.Ruminococcaceae	4.763		
22	Bacteria.Firmicutes.Erysipelotrichi.Erysipelotrichales.Erysipelotrichaceae.Clostridium.Clostridium_cocleatum	2.090		

23	Bacteria.Firmicutes.Bacilli.Lactobacillales.Lactobacillaceae.Lactobacillus	5.359		
24	Bacteria.Deferribacteres	4.715		
**25**	**Bacteria.Firmicutes.Erysipelotrichi.Erysipelotrichales**	**3.714**	**3.362 18**	**0 mg/kg PAP**
26	Bacteria.Cyanobacteria.Synechococcophycideae.Synechococcales	0.947		
27	Bacteria.Proteobacteria.Alphaproteobacteria.Sphingomonadales.Sphingomonadaceae.Sphingopyxis	0.872		
28	Bacteria.Firmicutes.Clostridia.Clostridiales.Ruminococcaceae.Butyricicoccus.Butyricicoccus_pullicaecorum	2.961		
29	Bacteria.Proteobacteria.Gammaproteobacteria.Pseudomonadales.Moraxellaceae.Acinetobacter.Acinetobacter_rh izosphaerae	2.059		
30	Bacteria.Acidobacteria.Sva0725.Sva0725	0.880		
31	Bacteria.Proteobacteria.Betaproteobacteria.Methylophilales.Methylophilaceae	1.345		
32	Bacteria.Firmicutes.Clostridia.Clostridiales.Ruminococcaceae.Butyricicoccus	2.961		
33	Bacteria.Proteobacteria.Deltaproteobacteria.Bdellovibrionales	0.903		
34	Bacteria.OD1	0.798		
35	Bacteria.Proteobacteria.Gammaproteobacteria.Pasteurellales	1.391		
36	Bacteria.Proteobacteria.Gammaproteobacteria.Enterobacteriales	2.056		
37	Bacteria.Synergistetes.Synergistia.Synergistales.Synergistaceae	0.965		
38	Bacteria.Proteobacteria.Gammaproteobacteria.Pseudomonadales.Moraxellaceae.Acinetobacter	2.513		
39	Bacteria.Bacteroidetes.Bacteroidia.Bacteroidales.Rikenellaceae.Alistipes.Alistipes_massiliensis	3.530		
**40**	**Bacteria.Firmicutes.Clostridia.Clostridiales.Ruminococcaceae.Ruminococcus**	**4.158**	**3.697 03**	**0 mg/kg PAP**
41	Bacteria.Bacteroidetes.Bacteroidia.Bacteroidales.Rikenellaceae.AF12	3.614		
42	Bacteria.Firmicutes.Clostridia.Clostridiales.Ruminococcaceae.Oscillospira	4.507		
43	Bacteria.Proteobacteria.Alphaproteobacteria.Rhizobiales	1.620		
44	Bacteria.Planctomycetes.Phycisphaerae.AKAU3564	0.823		
45	Bacteria.Planctomycetes.Phycisphaerae	0.823		
46	Bacteria.Proteobacteria.Alphaproteobacteria.Rhodospirillales.Acetobacteraceae	2.135		
47	Bacteria.Proteobacteria.Deltaproteobacteria.Desulfovibrionales	4.400		
48	Bacteria.Proteobacteria.Betaproteobacteria.Burkholderiales.Oxalobacteraceae	1.342		
**49**	**Bacteria.Proteobacteria.Epsilonproteobacteria**	**4.190**	**3.834 57**	**70 mg/kg CTX**

50	Bacteria.Proteobacteria.Gammaproteobacteria.Xanthomonadales.Xanthomonadaceae.Thermomonas.Thermomonas_fusca	1.830
51	Bacteria.Actinobacteria.Acidimicrobiia.Acidimicrobiales	0.903
52	Bacteria.Proteobacteria.Betaproteobacteria.Burkholderiales.Burkholderiaceae	1.148
53	Bacteria.Chlamydiae.Chlamydiia	0.903
54	Bacteria.Proteobacteria.Gammaproteobacteria.Enterobacteriales.Enterobacteriaceae.Serratia	1.139
55	Bacteria.Proteobacteria.Deltaproteobacteria.Myxococcales.Myxococcaceae.Anaeromyxobacter	1.573
56	Bacteria.Proteobacteria.Gammaproteobacteria.Legionellales	1.244
57	Bacteria.Deferribacteres.Deferribacteres.Deferribacterales.Deferribacteraceae	4.715
58	Bacteria.Actinobacteria.Actinobacteria.Actinomycetales.Intrasporangiaceae	1.139
59	Bacteria.Firmicutes.Bacilli.Lactobacillales.Lactobacillaceae.Pediococcus.Pediococcus_cellicola	1.424
60	Bacteria.Proteobacteria.Gammaproteobacteria.Enterobacteriales.Enterobacteriaceae.Pantoea	1.332
61	Bacteria.Proteobacteria.Betaproteobacteria.MND1	1.173
62	Bacteria.Verrucomicrobia.Verrucomicrobiae.Verrucomicrobiales.Verrucomicrobiaceae.Akkermansia.Akkermansia_muciniphila	2.563
63	Bacteria.Firmicutes.Bacilli.Bacillales.Staphylococcaceae.Staphylococcus.Staphylococcus_sciuri	2.213
64	Bacteria.Bacteroidetes.Bacteroidia.Bacteroidales.S24_7	5.533
65	Bacteria.Bacteroidetes.Bacteroidia.Bacteroidales.Rikenellaceae	5.241
66	Bacteria.Proteobacteria.Gammaproteobacteria.Pseudomonadales.Pseudomonadaceae	2.583
67	Bacteria.Proteobacteria.Gammaproteobacteria.Pseudomonadales.Pseudomonadaceae.Pseudomonas	2.583
68	Bacteria.Proteobacteria.Gammaproteobacteria.Alteromonadales.Alteromonadaceae.Marinobacter.Marinobacter_bryozoorum	1.144
69	Bacteria.Proteobacteria.Alphaproteobacteria.Rhizobiales.Bradyrhizobiaceae.Balneimonas	0.843
70	Bacteria.Firmicutes.Bacilli.Lactobacillales.Lactobacillaceae.Lactobacillus.Lactobacillus_salivarius	1.196
71	Bacteria.Actinobacteria.Actinobacteria.Bifidobacteriales.Bifidobacteriaceae.Bifidobacterium.Bifidobacterium_longum	1.521
72	Bacteria.Proteobacteria.Alphaproteobacteria.Rhizobiales.Rhizobiaceae.Agrobacterium	1.475
73	Bacteria.Proteobacteria.Gammaproteobacteria.Xanthomonadales.Xanthomonadaceae.Arenimonas	1.241
74	Bacteria.Proteobacteria.Betaproteobacteria.Procabacteriales	0.823
75	Bacteria.Planctomycetes.Phycisphaerae.Phycisphaerales	0.798
76	Bacteria.Proteobacteria.Alphaproteobacteria.Rhodospirillales.Rhodospirillaceae.Skermanella	0.965

77	Bacteria.Bacteroidetes.Bacteroidia.Bacteroidales.Bacteroidaceae.Bacteroides	4.458		
78	Bacteria.Firmicutes.Clostridia.Clostridiales._Mogibacteriaceae_	3.327		
79	Bacteria.Actinobacteria.Actinobacteria.Bifidobacteriales.Bifidobacteriaceae	2.351		
80	Bacteria.Proteobacteria.Gammaproteobacteria.Alteromonadales.Alteromonadaceae.HB2_32_21	1.901		
**81**	**Bacteria.Firmicutes.Bacilli.Lactobacillales.Streptococcaceae.Streptococcus**	**2.910**	**2.533 62**	**0 mg/kg PAP**
82	Bacteria.Chlamydiae.Chlamydiia.Chlamydiales.Chlamydiaceae.Chlamydia	0.903		
83	Bacteria.Actinobacteria.Rubrobacteria.Rubrobacterales.Rubrobacteraceae	0.872		
84	Bacteria.Bacteroidetes.Flavobacteriia.Flavobacteriales._Weeksellaceae_	0.798		
85	Bacteria.Proteobacteria.Betaproteobacteria.Rhodocyclales.Rhodocyclaceae.Dok59	1.099		
86	Bacteria.Firmicutes.Clostridia.Clostridiales.Lachnospiraceae.Roseburia.Roseburia_faecis	2.270		
87	Bacteria.Proteobacteria.Betaproteobacteria.Burkholderiales.Comamonadaceae.Aquabacterium	1.855		
88	Bacteria.Proteobacteria.Gammaproteobacteria.Pseudomonadales	2.850		
89	Bacteria.Firmicutes.Bacilli.Bacillales.Thermoactinomycetaceae.Thermoactinomyces.Thermoactinomyces_sanguinis	1.725		
90	Bacteria.Bacteroidetes.Bacteroidia.Bacteroidales._Paraprevotellaceae_.YRC22	1.148		
91	Bacteria.Spirochaetes.Spirochaetes	0.823		
92	Bacteria.Actinobacteria.Actinobacteria.Actinomycetales	2.293		
93	Bacteria.Firmicutes.Clostridia.Clostridiales.Lachnospiraceae.Dorea	2.494		
**94**	**Bacteria.Proteobacteria.Epsilonproteobacteria.Campylobacterales.Helicobacteraceae.Helicobacter**	**4.190**	**3.838 15**	**70 mg/kg CTX**
95	Bacteria.Firmicutes.Clostridia.Clostridiales.Christensenellaceae	1.924		
96	Bacteria.Proteobacteria.Alphaproteobacteria.Rhodospirillales.Rhodospirillaceae.Phaeospirillum.Phaeospirillum_fulvum	1.357		
97	Bacteria.Actinobacteria.Coriobacteriia.Coriobacteriales.Coriobacteriaceae.Adlercreutzia	3.691		
98	Bacteria.Spirochaetes	0.823		
99	Bacteria.Synergistetes.Synergistia	0.965		
100	Bacteria.Proteobacteria.Betaproteobacteria.Burkholderiales.Comamonadaceae	2.162		
101	Bacteria.Actinobacteria.Rubrobacteria.Rubrobacterales.Rubrobacteraceae.Rubrobacter	0.872		
102	Bacteria.Actinobacteria.Actinobacteria.Bifidobacteriales.Bifidobacteriaceae.Bifidobacterium	2.246		

**103**	**Bacteria.Proteobacteria.Epsilonproteobacteria.Campylobacterales.Helicobacteraceae**	**4.190**	**3.787 03**	**70 mg/kg CTX**
104	Bacteria.Proteobacteria.Betaproteobacteria.Rhodocyclales.Rhodocyclaceae.KD1_23	1.760		
105	Bacteria.Firmicutes.Clostridia.Clostridiales.Clostridiaceae.Clostridium.Clostridium_gasigenes	1.420		
**106**	**Bacteria.Firmicutes.Erysipelotrichi.Erysipelotrichales.Erysipelotrichaceae**	**3.714**	**3.378 76**	**0 mg/kg PAP**
107	Bacteria.Proteobacteria.Betaproteobacteria.Hydrogenophilales.Hydrogenophilaceae.Thiobacillus	2.313		
108	Bacteria.Proteobacteria.Gammaproteobacteria.Legionellales.Coxiellaceae.Rickettsiella	1.244		
109	Bacteria.Gemmatimonadetes.Gemm_1	1.090		
110	Bacteria.Firmicutes.Bacilli.Lactobacillales.Lactobacillaceae.Lactobacillus.Lactobacillus_zeae	2.292		
111	Bacteria.Proteobacteria.Gammaproteobacteria.Oceanospirillales.Halomonadaceae	1.683		
112	Bacteria.Chloroflexi.Thermomicrobia	1.350		
113	Bacteria.TM7.TM7_3	3.482		
114	Bacteria.Spirochaetes.Spirochaetes.Spirochaetales.Spirochaetaceae.Treponema	0.823		
115	Bacteria.Chloroflexi.Anaerolineae.Caldilineales.Caldilineaceae	0.998		
116	Bacteria.Firmicutes.Clostridia.Clostridiales.Ruminococcaceae.Gemmiger	0.789		
117	Bacteria.Firmicutes.Clostridia.Clostridiales.Lachnospiraceae._Ruminococcus_._Ruminococcus__torques	1.925		
118	Bacteria.Firmicutes.Bacilli.Lactobacillales.Streptococcaceae.Streptococcus.Streptococcus_alactolyticus	1.341		
119	Bacteria.Firmicutes.Clostridia.Clostridiales.Peptostreptococcaceae	1.341		
120	Bacteria.Spirochaetes.Spirochaetes.Spirochaetales	0.823		
121	Bacteria.Proteobacteria.Gammaproteobacteria.Oceanospirillales.Halomonadaceae.Halomonas.Halomonas_nitrit ophilus	1.174		
122	Bacteria.Verrucomicrobia	2.563		
123	Bacteria.TM7.TM7_3.CW040	3.482		
124	Bacteria.Proteobacteria.Alphaproteobacteria.Rhodospirillales.Acetobacteraceae.Acetobacter	2.135		
125	Bacteria.Firmicutes.Clostridia.Clostridiales.Lachnospiraceae.Clostridium.Clostridium_colinum	1.447		
126	Bacteria.Chlorobi.Ignavibacteria.Ignavibacteriales.Ignavibacteriaceae	0.947		
127	Bacteria.Proteobacteria.Deltaproteobacteria.Desulfovibrionales.Desulfovibrionaceae.Desulfovibrio.Desulfovibri o_C21_c20	4.344		
128	Bacteria.Proteobacteria.Gammaproteobacteria.Pseudomonadales.Moraxellaceae.Alkanindiges.Alkanindiges_illi noisensis	1.119		

129	Bacteria.Firmicutes.Bacilli.Lactobacillales.Aerococcaceae	2.238
130	Bacteria.Tenericutes.Mollicutes.Anaeroplasmatales	2.900
131	Bacteria.Proteobacteria.Epsilonproteobacteria.Campylobacterales.Helicobacteraceae.Flexispira	1.014
132	Bacteria.Chloroflexi.Anaerolineae.Ardenscatenales	1.099
133	Bacteria.Verrucomicrobia.Verrucomicrobiae.Verrucomicrobiales.Verrucomicrobiaceae	2.563
134	Bacteria.Proteobacteria	4.599
135	Bacteria.Actinobacteria.Actinobacteria.Actinomycetales.Microbacteriaceae	1.776
136	Bacteria.Bacteroidetes.Bacteroidia.Bacteroidales.Bacteroidaceae	4.458
137	Bacteria.Proteobacteria.Gammaproteobacteria.Xanthomonadales.Xanthomonadaceae.Stenotrophomonas	1.196
138	Bacteria.OD1.ABY1	0.798
139	Bacteria.Actinobacteria.Acidimicrobiia.Acidimicrobiales.C111	0.903
140	Bacteria.Chloroflexi.Anaerolineae.Ardenscatenales.Ardenscatenaceae	1.099
141	Bacteria.Proteobacteria.Alphaproteobacteria.Rhizobiales.Rhizobiaceae	1.475
142	Bacteria.Proteobacteria.Alphaproteobacteria.Rhodobacterales	1.119
143	Bacteria.Proteobacteria.Alphaproteobacteria.Rhizobiales.Bradyrhizobiaceae	1.144
144	Bacteria.Proteobacteria.Alphaproteobacteria.Sphingomonadales.Sphingomonadaceae.Kaistobacter	1.204
145	Bacteria.Proteobacteria.Betaproteobacteria.Nitrosomonadales	1.099
146	Bacteria.Proteobacteria.Betaproteobacteria.Burkholderiales.Alcaligenaceae	2.879
147	Bacteria.Firmicutes.Bacilli.Lactobacillales.Enterococcaceae.Enterococcus.Enterococcus_sulfureus	0.823
148	Bacteria.Proteobacteria.Alphaproteobacteria.Rhodobacterales.Hyphomonadaceae	0.798
149	Bacteria.Bacteroidetes.Bacteroidia.Bacteroidales.Rikenellaceae.Alistipes.Alistipes_putredinis	1.014
150	Bacteria.Proteobacteria.Alphaproteobacteria.Rhodospirillales.Rhodospirillaceae.Dongia.Dongia_mobilis	1.315
151	Bacteria.Verrucomicrobia.Verruco_5	0.903
152	Bacteria.Proteobacteria.Betaproteobacteria.Burkholderiales.Comamonadaceae.Rubrivivax.Rubrivivax_gelatinosus	1.486
153	Bacteria.Bacteroidetes.Bacteroidia.Bacteroidales.Rikenellaceae.Alistipes.Alistipes_indistinctus	2.897
154	Bacteria.Proteobacteria.Alphaproteobacteria.Rhizobiales.Brucellaceae	0.950
155	Bacteria.Chloroflexi.Anaerolineae.Caldilineales	0.998
156	Bacteria.OP8.OP8_1	1.119
157	Bacteria.Proteobacteria.Deltaproteobacteria.Bdellovibrionales.Bacteriovoracaceae.Peredibacter	0.903

158	Bacteria.Firmicutes.Bacilli.Bacillales.Staphylococcaceae.Jeotgalicoccus.Jeotgalicoccus_psychrophilus	2.098		
159	Bacteria.Firmicutes.Clostridia.Clostridiales.Lachnospiraceae.Lactonifactor.Lactonifactor_longoviformis	0.943		
160	Bacteria.Proteobacteria.Gammaproteobacteria.Xanthomonadales.Xanthomonadaceae	1.897		
161	Bacteria.Tenericutes.Mollicutes.RF39	2.779		
162	Bacteria.Tenericutes.RF3.ML615J_28	0.774		
163	Bacteria.Cyanobacteria.4C0d_2.YS2	2.091		
164	Bacteria.Firmicutes.Clostridia.Clostridiales.Ruminococcaceae.Faecalibacterium.Faecalibacterium_prausnitzii	2.405		
165	Bacteria.Tenericutes.RF3	0.774		
**166**	**Bacteria.Firmicutes.Clostridia.Clostridiales.Lachnospiraceae.Clostridium**	**3.201**	**2.703 63**	**CTX+ 6 mg/kg LEP**
167	Bacteria.Proteobacteria.Gammaproteobacteria.Xanthomonadales.Xanthomonadaceae.Arenimonas.Arenimonas_ oryziterrae	1.241		
168	Bacteria.Proteobacteria.Betaproteobacteria.Methylophilales	1.345		
169	Bacteria.Proteobacteria.Gammaproteobacteria.Xanthomonadales	1.995		
170	Bacteria.Firmicutes.Clostridia.Clostridiales.Lachnospiraceae.Roseburia	3.285		
171	Bacteria.Proteobacteria.Alphaproteobacteria.Rhodobacterales.Rhodobacteraceae	1.119		
172	Bacteria.Firmicutes.Clostridia.Clostridiales.Clostridiaceae.Clostridium.Clostridium_perfringens	1.578		
173	Bacteria.Proteobacteria.TA18	0.823		
174	Bacteria.Acidobacteria._Chloracidobacteria_.RB41	0.798		
175	Bacteria.Proteobacteria.Alphaproteobacteria	2.300		
176	Bacteria.Proteobacteria.Alphaproteobacteria.Rhodospirillales.Rhodospirillaceae	1.756		
177	Bacteria.Proteobacteria.Alphaproteobacteria.Rhizobiales.Xanthobacteraceae	1.014		
178	Bacteria.Actinobacteria.Acidimicrobiia	0.903		
**179**	**Bacteria.Chloroflexi.Anaerolineae.SBR1031**	**2.030**	**2.738 23**	**70 mg/kg CTX**
180	Bacteria.Firmicutes.Bacilli.Bacillales.Bacillaceae	1.686		
181	Bacteria.Proteobacteria.Deltaproteobacteria.Bdellovibrionales.Bacteriovoracaceae	0.903		
182	Bacteria.Proteobacteria.Betaproteobacteria	2.935		

183	Bacteria.Proteobacteria.Deltaproteobacteria.Desulfovibrionales.Desulfovibrionaceae.Desulfovibrio.Desulfovibrio_alaskensis	1.267		
184	Bacteria.Actinobacteria.Actinobacteria.Actinomycetales.Microbacteriaceae.Cryocola	1.776		
185	Bacteria.Chloroflexi.Anaerolineae.S0208	0.789		
186	Bacteria.Proteobacteria.Alphaproteobacteria.Caulobacterales.Caulobacteraceae	1.048		
187	Bacteria.Bacteroidetes.Bacteroidia.Bacteroidales._Paraprevotellaceae_.CF231	1.172		
188	Bacteria.Proteobacteria.Alphaproteobacteria.Rhizobiales.Xanthobacteraceae.Labrys	1.014		
189	Bacteria.Proteobacteria.Betaproteobacteria.Ellin6067	1.320		
190	Bacteria.Firmicutes.Clostridia.Clostridiales.Veillonellaceae.Dialister	0.798		
**191**	**Bacteria.Chloroflexi.Anaerolineae.SBR1031.A4b**	**1.409**	**3.300 24**	**70 mg/kg CTX**
192	Bacteria.Actinobacteria.Thermoleophilia.Gaiellales.Gaiellaceae	0.872		
193	Bacteria.Gemmatimonadetes.Gemmatimonadetes.Gemmatimonadales.Ellin5301	0.798		
194	Bacteria.Firmicutes	5.756		
195	Bacteria.Firmicutes.Clostridia.Clostridiales.Lachnospiraceae.Blautia	2.445		
196	Bacteria.Planctomycetes._Brocadiae_.Brocadiales.Brocadiaceae	1.673		
197	Bacteria.Chloroflexi.S085	0.867		
198	Bacteria.Cyanobacteria.Synechococcophycideae.Synechococcales.Synechococcaceae.Synechococcus	0.947		
199	Bacteria.Proteobacteria.Alphaproteobacteria.Caulobacterales	1.048		
200	Bacteria.Firmicutes.Clostridia.Clostridiales.Lachnospiraceae	5.282		
201	Bacteria.Bacteroidetes.Bacteroidia.Bacteroidales.Porphyromonadaceae.Parabacteroides	2.572		
202	Bacteria.Proteobacteria.Gammaproteobacteria.Alteromonadales	1.901		
203	Bacteria.Firmicutes.Bacilli.Bacillales.Planococcaceae.Staphylococcus.Staphylococcus_saprophyticus	1.604		
204	Bacteria.Proteobacteria.Gammaproteobacteria.Xanthomonadales.Sinobacteraceae	1.300		
205	Bacteria.Deferribacteres.Deferribacteres.Deferribacterales.Deferribacteraceae.Mucispirillum.Mucispirillum_schaedleri	4.715		
206	Bacteria.Chlamydiae.Chlamydiia.Chlamydiales.Chlamydiaceae	0.903		
207	Bacteria.Proteobacteria.Betaproteobacteria.Nitrosomonadales.Nitrosomonadaceae.Nitrosomonas.Nitrosomonas_europaea	1.099		
208	Bacteria.Proteobacteria.Deltaproteobacteria.Myxococcales.Haliangiaceae	0.873		
209	Bacteria.Chlamydiae	0.903		

210	Bacteria.Bacteroidetes.Flavobacteriia	0.798		
211	Bacteria.Cyanobacteria.ML635J_21	0.880		
212	Bacteria.Bacteroidetes.Bacteroidia.Bacteroidales.Bacteroidaceae.Bacteroides.Bacteroides_uniformis	1.265		
213	Bacteria.Planctomycetes.Planctomycetia	1.111		
214	Bacteria.Proteobacteria.Gammaproteobacteria.Enterobacteriales.Enterobacteriaceae	2.056		
215	Bacteria.Cyanobacteria.Synechococcophycideae	0.947		
216	Bacteria.Firmicutes.Bacilli.Bacillales.Staphylococcaceae.Staphylococcus	3.313		
217	Bacteria.Deferribacteres.Deferribacteres	4.715		
218	Bacteria.Actinobacteria.Actinobacteria.Bifidobacteriales	2.351		
219	Bacteria.Acidobacteria.Acidobacteria_6.iii1_15	1.123		
220	Bacteria.TM7.TM7_3.CW040.F16	3.482		
221	Bacteria.Proteobacteria.Deltaproteobacteria.Myxococcales.Myxococcaceae	1.573		
222	Bacteria.Actinobacteria.Coriobacteriia	3.711		
223	Bacteria.Proteobacteria.Deltaproteobacteria.Desulfovibrionales.Desulfovibrionaceae.Desulfovibrio	4.387		
224	Bacteria.Bacteroidetes.Flavobacteriia.Flavobacteriales._Weeksellaceae_.Chryseobacterium	0.798		
225	Bacteria	6.000		
226	Bacteria.Cyanobacteria.4C0d_2	2.091		
227	Bacteria.Gemmatimonadetes	1.336		
228	Bacteria.Proteobacteria.Gammaproteobacteria.Xanthomonadales.Xanthomonadaceae.Pseudoxanthomonas.Pseudoxanthomonas_mexicana	1.056		
229	Bacteria.Proteobacteria.Deltaproteobacteria	4.400		
**230**	**Bacteria.Bacteroidetes.Bacteroidia.Bacteroidales._Paraprevotellaceae_.Paraprevotella**	**2.711**	**2.460<br>90**	**CTX+<br>100 mg/kg<br>PAP**
231	Bacteria.Proteobacteria.Gammaproteobacteria.Alteromonadales.Alteromonadaceae.Marinobacter	1.144		
232	Bacteria.Proteobacteria.Gammaproteobacteria.Xanthomonadales.Xanthomonadaceae.Aquimonas	1.827		
233	Bacteria.Bacteroidetes.Bacteroidia.Bacteroidales._Odoribacteraceae_	4.185	3.653<br>41	**0 mg/kg<br>PAP**
234	Bacteria.Proteobacteria.Gammaproteobacteria.Pseudomonadales.Moraxellaceae.Perlucidibaca	1.952	2.410<br>40	**0 mg/kg<br>PAP**
235	Bacteria.Proteobacteria.Betaproteobacteria.Nitrosomonadales.Nitrosomonadaceae	1.099		

236	Bacteria.Proteobacteria.Gammaproteobacteria.Pasteurellales.Pasteurellaceae.Aggregatibacter	1.391		
237	Bacteria.Bacteroidetes.Cytophagia.Cytophagales	0.947		
238	Bacteria.Chloroflexi.Thermomicrobia.JG30_KF_CM45	1.350		
239	Bacteria.Bacteroidetes.Bacteroidia.Bacteroidales.Porphyromonadaceae.Parabacteroides.Parabacteroides_distasonis	2.037		
240	Bacteria.Planctomycetes._Brocadiae_.Brocadiales	1.673		
241	Bacteria.Firmicutes.Clostridia.Clostridiales.Clostridiaceae	3.161		
242	Bacteria.Firmicutes.Clostridia.Clostridiales.Veillonellaceae.Phascolarctobacterium	1.549		
243	Bacteria.Proteobacteria.Deltaproteobacteria.NB1_j	1.044		
244	Bacteria.Proteobacteria.Deltaproteobacteria.Bdellovibrionales.Bacteriovoracaceae.Peredibacter.Peredibacter_starrii	0.903		
**245**	**Bacteria.Firmicutes.Clostridia.Clostridiales.Ruminococcaceae.Ruminococcus.Ruminococcus_albus**	**1.545**	**2.950 26**	**CTX+ 6 mg/kg LEP**
246	Bacteria.Bacteroidetes.Bacteroidia.Bacteroidales._Paraprevotellaceae_._Prevotella_	1.056		
247	Bacteria.Firmicutes.Bacilli.Bacillales.Staphylococcaceae	3.329		
248	Bacteria.Proteobacteria.Betaproteobacteria.Methylophilales.Methylophilaceae.Methylotenera.Methylotenera_versatilis	1.345		
249	Bacteria.Proteobacteria.Betaproteobacteria.Methylophilales.Methylophilaceae.Methylotenera	1.345		
250	Bacteria.Proteobacteria.Betaproteobacteria.Rhodocyclales	2.430		
251	Bacteria.Actinobacteria.Actinobacteria.Actinomycetales.Mycobacteriaceae	1.748		
252	Bacteria.Planctomycetes._Brocadiae_.Brocadiales.Brocadiaceae.Candidatus_Brocadia	1.673		
253	Bacteria.Proteobacteria.Gammaproteobacteria	3.027		
254	Bacteria.Actinobacteria.Actinobacteria	2.475		
255	Bacteria.Proteobacteria.Betaproteobacteria.Burkholderiales.Oxalobacteraceae.Ralstonia	1.014		
256	Bacteria.Actinobacteria.Actinobacteria.Actinomycetales.Cellulomonadaceae	1.014		
257	Bacteria.Firmicutes.Clostridia.Clostridiales.Veillonellaceae.Selenomonas	1.679		
258	Bacteria.Proteobacteria.Alphaproteobacteria.Rhodospirillales.Rhodospirillaceae.Dongia	1.315		
259	Bacteria.Acidobacteria.Acidobacteria_6.CCU21	0.838		
260	Bacteria.Firmicutes.Bacilli.Lactobacillales.Enterococcaceae.Enterococcus	3.327		

261	Bacteria.Chlorobi.OPB56	1.048		
262	Bacteria.Verrucomicrobia.Verrucomicrobiae.Verrucomicrobiales.Verrucomicrobiaceae.Akkermansia	2.563		
263	Bacteria.TM7.TM7_1	1.023		
264	Bacteria.Proteobacteria.Betaproteobacteria.Hydrogenophilales.Hydrogenophilaceae	2.313		
265	Bacteria.Proteobacteria.Gammaproteobacteria.Oceanospirillales.Oceanospirillaceae	1.283		
266	Bacteria.Firmicutes.Clostridia.Clostridiales.Veillonellaceae.Selenomonas.Selenomonas_lacticifex	1.679		
267	Bacteria.Synergistetes.Synergistia.Synergistales	0.965		
268	Bacteria.TM7	3.484		
269	Bacteria.Actinobacteria.Actinobacteria.Actinomycetales.Nocardioidaceae.Marmoricola	1.245		
270	Bacteria.Firmicutes.Clostridia.Clostridiales.Lachnospiraceae._Ruminococcus_	3.848		
271	Bacteria.Proteobacteria.Gammaproteobacteria.Xanthomonadales.Xanthomonadaceae.Thermomonas	1.830		
272	Bacteria.Planctomycetes.Planctomycetia.Gemmatales	1.111		
273	Bacteria.Proteobacteria.Gammaproteobacteria.Chromatiales	1.755		
274	Bacteria.Proteobacteria.Alphaproteobacteria.Rhodospirillales.Rhodospirillaceae.Phaeospirillum	1.357		
275	Bacteria.Bacteroidetes.Bacteroidia.Bacteroidales._Barnesiellaceae_.Barnesiella	1.197		
276	Bacteria.Firmicutes.Clostridia.Clostridiales.Ruminococcaceae.Ruminococcus.Ruminococcus_bromii	1.921		
**277**	**Bacteria.Firmicutes.Erysipelotrichi**	**3.714**	**3.365 31**	**0 mg/kg PAP**
278	Bacteria.Actinobacteria.Coriobacteriia.Coriobacteriales.Coriobacteriaceae	3.711		
279	Bacteria.Firmicutes.Erysipelotrichi.Erysipelotrichales.Erysipelotrichaceae.Coprobacillus	1.241		
280	Bacteria.Actinobacteria.KIST_JJY010	0.823		
281	Bacteria.Proteobacteria.Alphaproteobacteria.Rhodospirillales.Rhodospirillaceae.Azospirillum	1.534		
282	Bacteria.Bacteroidetes.Bacteroidia.Bacteroidales.p_2534_18B5	1.144		
283	Bacteria.Tenericutes.Mollicutes.Anaeroplasmatales.Anaeroplasmataceae	2.900		
284	Bacteria.Chloroflexi.Ktedonobacteria.Thermogemmatisporales.Thermogemmatisporaceae	0.822		
285	Bacteria.Cyanobacteria	2.121		
286	Bacteria.Proteobacteria.Gammaproteobacteria.Alteromonadales.Alteromonadaceae	1.901		
287	Bacteria.Proteobacteria.Gammaproteobacteria.Pseudomonadales.Moraxellaceae	2.626		
288	Bacteria.Bacteroidetes.Bacteroidia.Bacteroidales.Porphyromonadaceae.Parabacteroides.Parabacteroides_gordonii	0.646		

289	Bacteria.Proteobacteria.Alphaproteobacteria.Sphingomonadales.Sphingomonadaceae	1.524		
290	Bacteria.Cyanobacteria.Chloroplast.Streptophyta	1.666		
291	Bacteria.Firmicutes.Clostridia.Clostridiales.Ruminococcaceae.Faecalibacterium	2.427		
292	Bacteria.Firmicutes.Clostridia.Clostridiales.Lachnospiraceae._Ruminococcus_._Ruminococcus__gnavus	3.848		
293	Bacteria.Proteobacteria.Betaproteobacteria.Nitrosomonadales.Nitrosomonadaceae.Nitrosomonas	1.099		
294	Bacteria.Acidobacteria._Chloracidobacteria_.11_24	1.204		
295	Bacteria.Bacteroidetes.Bacteroidia.Bacteroidales	5.706		
296	Bacteria.Bacteroidetes.Bacteroidia.Bacteroidales.Rikenellaceae.Blvii28	1.528		
297	Bacteria.Cyanobacteria.Synechococcophycideae.Synechococcales.Synechococcaceae	0.947		
298	Bacteria.Firmicutes.Bacilli.Lactobacillales.Leuconostocaceae	1.049		
299	Bacteria.Firmicutes.Clostridia.Clostridiales.Ruminococcaceae.Clostridium	1.556		
300	Bacteria.Bacteroidetes.Bacteroidia.Bacteroidales.Marinilabiaceae	1.044		
301	Bacteria.Firmicutes.Bacilli.Bacillales.Thermoactinomycetaceae.Thermoactinomyces	1.725		
302	Bacteria.Proteobacteria.Gammaproteobacteria.Pasteurellales.Pasteurellaceae	1.391		
303	Bacteria.Verrucomicrobia.Verruco_5.WCHB1_41	0.903		
304	Bacteria.Firmicutes.Clostridia.Clostridiales.Lachnospiraceae.Dorea.Dorea_longicatena	0.843		
305	Bacteria.Firmicutes.Clostridia.Clostridiales.Peptococcaceae	2.744		
306	Bacteria.Actinobacteria.Rubrobacteria	0.872		
307	Bacteria.Firmicutes.Bacilli.Lactobacillales.Enterococcaceae	3.329		
308	Bacteria.Proteobacteria.Gammaproteobacteria.Pasteurellales.Pasteurellaceae.Aggregatibacter.Aggregatibacter_pneumotropica	1.391		
309	Bacteria.Actinobacteria.Actinobacteria.Actinomycetales.Micrococcaceae	1.792		
**310**	**Bacteria.Bacteroidetes.Bacteroidia.Bacteroidales._Paraprevotellaceae_**	**2.711**	**2.43953**	**CTX+ 100 mg/kg PAP**
311	Bacteria.Firmicutes.Clostridia.Clostridiales.Ruminococcaceae.Gemmiger.Gemmiger_formicilis	0.789		
312	Bacteria.Firmicutes.Bacilli.Lactobacillales.Lactobacillaceae.Lactobacillus.Lactobacillus_pontis	1.511		
**313**	**Bacteria.Proteobacteria.Epsilonproteobacteria.Campylobacterales**	**4.190**	**3.83568**	**70 mg/kg CTX**
314	Bacteria.Proteobacteria.Gammaproteobacteria.Oceanospirillales.Halomonadaceae.Halomonas	1.683		

315	Bacteria.Acidobacteria.Acidobacteria_6	1.123
316	Bacteria.Proteobacteria.Gammaproteobacteria.Legionellales.Legionellaceae	0.903
317	Bacteria.Firmicutes.Bacilli.Lactobacillales.Lactobacillaceae.Pediococcus	1.721
318	Bacteria.Firmicutes.Bacilli	5.376
319	Bacteria.Firmicutes.Erysipelotrichi.Erysipelotrichales.Erysipelotrichaceae.cc_115	2.057
320	Bacteria.Firmicutes.Bacilli.Bacillales.Staphylococcaceae.Jeotgalicoccus	2.098
321	Bacteria.Planctomycetes._Brocadiae_	1.673
322	Bacteria.Proteobacteria.Alphaproteobacteria.Rhizobiales.Rhizobiaceae.Rhizobium	1.099
323	Bacteria.Proteobacteria.Alphaproteobacteria.Sphingomonadales	1.524
324	Bacteria.Proteobacteria.Alphaproteobacteria.Sphingomonadales.Sphingomonadaceae.Novosphingobium	1.350
325	Bacteria.Bacteroidetes.Bacteroidia.Bacteroidales.Prevotellaceae	3.585
326	Bacteria.Tenericutes	3.091
327	Bacteria.Proteobacteria.Deltaproteobacteria.Myxococcales	1.573
328	Bacteria.Proteobacteria.Betaproteobacteria.Burkholderiales.Burkholderiaceae.Burkholderia	0.965
329	Bacteria.Proteobacteria.Betaproteobacteria.Burkholderiales.Comamonadaceae.Ramlibacter	1.236
330	Bacteria.Actinobacteria.Thermoleophilia	0.872
331	Bacteria.Proteobacteria.Betaproteobacteria.Burkholderiales.Comamonadaceae.Comamonas	1.596
332	Bacteria.Bacteroidetes.Bacteroidia.Bacteroidales.Rikenellaceae.Alistipes.Alistipes_onderdonkii	1.646
333	Bacteria.Proteobacteria.Gammaproteobacteria.Oceanospirillales	1.829
334	Bacteria.Firmicutes.Clostridia.Clostridiales	5.606
335	Bacteria.OP8	1.119
336	Bacteria.Firmicutes.Bacilli.Gemellales	1.701
337	Bacteria.Firmicutes.Clostridia.Clostridiales.Ruminococcaceae.Clostridium.Clostridium_methylpentosum	1.556
338	Bacteria.Actinobacteria.Coriobacteriia.Coriobacteriales	3.711
339	Bacteria.Firmicutes.Bacilli.Lactobacillales.Streptococcaceae.Lactococcus	3.548
340	Bacteria.Gemmatimonadetes.Gemmatimonadetes.Gemmatimonadales	0.798
341	Bacteria.Proteobacteria.Betaproteobacteria.Burkholderiales.Burkholderiaceae.Burkholderia.Burkholderia_gladioli	0.965
342	Bacteria.Actinobacteria.Rubrobacteria.Rubrobacterales	0.872
343	Bacteria.Bacteroidetes.Bacteroidia.Bacteroidales._Barnesiellaceae_.Barnesiella.Barnesiella_intestinihominis	1.197

344	Bacteria.Chloroflexi.TK10.B07_WMSP1	0.843		
345	Bacteria.Firmicutes.Clostridia.Clostridiales.Ruminococcaceae.Ruminococcus.Ruminococcus_callidus	1.204		
346	Bacteria.Actinobacteria.Actinobacteria.Bifidobacteriales.Bifidobacteriaceae.Gardnerella	1.683		
347	Bacteria.Proteobacteria.Gammaproteobacteria.Legionellales.Coxiellaceae	1.244		
348	Bacteria.Tenericutes.Mollicutes.Anaeroplasmatales.Anaeroplasmataceae.Anaeroplasma	2.900		
349	Bacteria.Proteobacteria.Gammaproteobacteria.Pseudomonadales.Moraxellaceae.Alkanindiges	1.119		
350	Bacteria.Proteobacteria.Betaproteobacteria.Burkholderiales.Comamonadaceae.Rubrivivax	1.486		
351	Bacteria.Chlorobi.Ignavibacteria	0.947		
352	Bacteria.Bacteroidetes.Cytophagia	0.947		
353	Bacteria.Proteobacteria.Betaproteobacteria.Procabacteriales.Procabacteriaceae	0.823		
**354**	**Bacteria.Chloroflexi.Anaerolineae.SBR1031.SHA_31**	**1.911**	**2.440 34**	**70 mg/kg CTX**
355	Bacteria.Firmicutes.Erysipelotrichi.Erysipelotrichales.Erysipelotrichaceae.Allobaculum	0.872		
356	Bacteria.Proteobacteria.Alphaproteobacteria.Rhodospirillales	2.169		
357	Bacteria.Acidobacteria	1.373		
358	Bacteria.Actinobacteria.Actinobacteria.Actinomycetales.Nocardioidaceae.Nocardioides	0.873		
359	Bacteria.Firmicutes.Bacilli.Bacillales	3.362		
360	Bacteria.Firmicutes.Clostridia.Clostridiales.Lachnospiraceae.Shuttleworthia	1.474		
361	Bacteria.Bacteroidetes.Flavobacteriia.Flavobacteriales	0.798		
362	Bacteria.Firmicutes.Clostridia.Clostridiales.Ruminococcaceae.Ruminococcus.Ruminococcus_flavefaciens	2.041		
363	Bacteria.Spirochaetes.Spirochaetes.Spirochaetales.Spirochaetaceae	0.823		
364	Bacteria.Bacteroidetes.Bacteroidia.Bacteroidales._Barnesiellaceae_	1.197		
365	Bacteria.Actinobacteria.Coriobacteriia.Coriobacteriales.Coriobacteriaceae.Eggerthella	0.847		
366	Bacteria.Chloroflexi.Ktedonobacteria.Thermogemmatisporales	0.822		
367	Bacteria.Proteobacteria.Betaproteobacteria.Rhodocyclales.Rhodocyclaceae	2.430		
368	Bacteria.Proteobacteria.Gammaproteobacteria.Enterobacteriales.Enterobacteriaceae.Shigella	1.978		
369	Bacteria.Proteobacteria.Betaproteobacteria.Hydrogenophilales	2.313		
370	Bacteria.Proteobacteria.Deltaproteobacteria.Desulfovibrionales.Desulfovibrionaceae.Bilophila	2.890		
371	Bacteria.Chloroflexi.TK10	0.843		
372	Bacteria.Chlorobi	1.048		

373	Bacteria.Bacteroidetes.Bacteroidia	5.706		
374	Bacteria.Firmicutes.Bacilli.Bacillales.Thermoactinomycetaceae	1.725		
375	Bacteria.Proteobacteria.Gammaproteobacteria.Pseudomonadales.Moraxellaceae.Acinetobacter.Acinetobacter_guillouiae	2.432		
376	Bacteria.Bacteroidetes.Bacteroidia.Bacteroidales.Prevotellaceae.Prevotella	3.584		
377	Bacteria.Proteobacteria.Betaproteobacteria.Procabacteriales.Procabacteriaceae.Procabacter	0.823		
378	Bacteria.Firmicutes.Clostridia.Clostridiales.Eubacteriaceae.Anaerofustis	1.270		
379	Bacteria.Firmicutes.Bacilli.Lactobacillales.Lactobacillaceae.Lactobacillus.Lactobacillus_vaginalis	4.125		
380	Bacteria.Proteobacteria.Gammaproteobacteria.Chromatiales.Ectothiorhodospiraceae	1.755		
381	Bacteria.Verrucomicrobia.Verruco_5.WCHB1_41.RFP12	0.903		
382	Bacteria.Actinobacteria.Thermoleophilia.Gaiellales	0.872		
383	Bacteria.Bacteroidetes.Bacteroidia.Bacteroidales.Porphyromonadaceae.Macellibacteroides	0.798		
384	Bacteria.Proteobacteria.Gammaproteobacteria.Xanthomonadales.Xanthomonadaceae.Pseudoxanthomonas	1.056		
385	Bacteria.Firmicutes.Bacilli.Lactobacillales	5.372		
386	Bacteria.Firmicutes.Bacilli.Lactobacillales.Aerococcaceae.Atopostipes.Atopostipes_suicloacalis	2.196		
387	Bacteria.Proteobacteria.Betaproteobacteria.Rhodocyclales.Rhodocyclaceae.Zoogloea	2.193		
388	Bacteria.Gemmatimonadetes.Gemmatimonadetes	0.798		
**389**	**Bacteria.Proteobacteria.Gammaproteobacteria.Alteromonadales.Idiomarinaceae**	**1.720**	**2.773 53**	**CTX+ 100 mg/kg PAP**
390	Bacteria.Firmicutes.Clostridia.Clostridiales.Lachnospiraceae.Lactonifactor	0.943		
391	Bacteria.Cyanobacteria.Chloroplast	1.666		
392	Bacteria.Gemmatimonadetes.Gemm_2	0.774		
393	Bacteria.Firmicutes.Bacilli.Lactobacillales.Lactobacillaceae	5.360		
394	Bacteria.Proteobacteria.Deltaproteobacteria.Desulfovibrionales.Desulfovibrionaceae	4.400		
395	Bacteria.Chloroflexi	2.102		
396	Bacteria.Firmicutes.Bacilli.Lactobacillales.Lactobacillaceae.Lactobacillus.Lactobacillus_helveticus	1.602		
397	Bacteria.Firmicutes.Clostridia.Clostridiales.Ruminococcaceae.Oscillospira.Oscillospira_guilliermondii	0.872		
398	Bacteria.Bacteroidetes.Bacteroidia.Bacteroidales.Rikenellaceae.Alistipes	3.643		
399	Bacteria.Firmicutes.Clostridia.Clostridiales.Veillonellaceae	1.946		

400	Bacteria.Proteobacteria.Gammaproteobacteria.HOC36	0.880		
401	Bacteria.Chlamydiae.Chlamydiia.Chlamydiales	0.903		
402	Bacteria.Verrucomicrobia.Verrucomicrobiae	2.563		
403	Bacteria.Firmicutes.Clostridia	5.606		
404	Bacteria.Actinobacteria.Actinobacteria.Actinomycetales.Cellulomonadaceae.Cellulomonas	1.014		
405	Bacteria.Firmicutes.Clostridia.Clostridiales.Lachnospiraceae.Coprococcus	3.609		
406	Bacteria.Firmicutes.Erysipelotrichi.Erysipelotrichales.Erysipelotrichaceae.Clostridium	2.090		
407	Bacteria.Synergistetes	0.965		
408	Bacteria.Firmicutes.Bacilli.Bacillales.Planococcaceae.Sporosarcina	1.497		
409	Bacteria.Firmicutes.Clostridia.Clostridiales.Clostridiaceae.Clostridium	3.114		
410	Bacteria.Proteobacteria.Gammaproteobacteria.Legionellales.Legionellaceae.Legionella	0.903		
411	Bacteria.Chloroflexi.Anaerolineae.SBR1031.SJA_101	0.998		
412	Bacteria.Bacteroidetes.Cytophagia.Cytophagales.Cytophagaceae	0.947		
413	Bacteria.Proteobacteria.Alphaproteobacteria.Rhizobiales.Phyllobacteriaceae.Chelativorans	0.943		
414	Bacteria.Planctomycetes	1.822		
415	Bacteria.Bacteroidetes.Bacteroidia.Bacteroidales.Rikenellaceae.Alistipes.Alistipes_finegoldii	0.947		
416	Bacteria.WPS_2	1.449		
417	Bacteria.Proteobacteria.TA18.CV90	0.823		
418	Bacteria.Actinobacteria.Actinobacteria.Actinomycetales.Nocardioidaceae	1.398		
419	Bacteria.Bacteroidetes.Bacteroidia.Bacteroidales.BS11	1.221		
**420**	**Bacteria.Bacteroidetes.Bacteroidia.Bacteroidales._Odoribacteraceae_.Odoribacter**	**4.185**	**3.623 11**	**0 mg/kg PAP**
421	Bacteria.Proteobacteria.Betaproteobacteria.SC_I_84	1.075		
422	Bacteria.Firmicutes.Bacilli.Lactobacillales.Leuconostocaceae.Leuconostoc	1.049		
423	Bacteria.Verrucomicrobia.Verrucomicrobiae.Verrucomicrobiales	2.563		
424	Bacteria.Deferribacteres.Deferribacteres.Deferribacterales.Deferribacteraceae.Mucispirillum	4.715		
425	Bacteria.Proteobacteria.Alphaproteobacteria.Caulobacterales.Caulobacteraceae.Phenylobacterium	1.048		
426	Bacteria.Tenericutes.Mollicutes	3.091		
427	Bacteria.Bacteroidetes.Bacteroidia.Bacteroidales.Bacteroidaceae.Bacteroides.Bacteroides_acidifaciens	4.292		
428	Bacteria.Proteobacteria.Betaproteobacteria.Burkholderiales	2.901		

429	Bacteria.Firmicutes.Bacilli.Bacillales.Planococcaceae.Staphylococcus	1.604		
430	Bacteria.Proteobacteria.Betaproteobacteria.Burkholderiales.Alcaligenaceae.Sutterella	2.879		
431	Bacteria.Firmicutes.Clostridia.Clostridiales.Ruminococcaceae.Anaerotruncus	2.948		
432	Bacteria.Proteobacteria.Betaproteobacteria.Burkholderiales.Comamonadaceae.Hydrogenophaga	0.789		
433	Bacteria.Chlorobi.Ignavibacteria.Ignavibacteriales	0.947		
434	Bacteria.Chloroflexi.Ktedonobacteria	0.822		
435	Bacteria.Deferribacteres.Deferribacteres.Deferribacterales	4.715		
436	Bacteria.Chloroflexi.Gitt_GS_136	1.139		
437	Bacteria.Firmicutes.Bacilli.Lactobacillales.Aerococcaceae.Atopostipes	2.196		
438	Bacteria.Firmicutes.Clostridia.Clostridiales.Veillonellaceae.Megasphaera	0.872		
439	Bacteria.Chloroflexi.Anaerolineae.GCA004	1.289		
440	Bacteria.Bacteroidetes.Bacteroidia.Bacteroidales.Porphyromonadaceae	2.576		
441	Bacteria.Actinobacteria	3.730		
442	Bacteria.Actinobacteria.Actinobacteria.Actinomycetales.Mycobacteriaceae.Mycobacterium	1.748		
443	Bacteria.Planctomycetes.Planctomycetia.Gemmatales.Gemmataceae	1.111		
**444**	**Bacteria.Chloroflexi.Anaerolineae**	**2.078**	**2.537 82**	**70 mg/kg CTX**

Ctrl, control, CTX, cyclophosphamide; PAPS1, water-soluble PA polysaccharides, LNT, lentinan.
